# Development of a Numerical Model of a Bio-Inspired Sea Lion Robot

**DOI:** 10.3390/biomimetics10110772

**Published:** 2025-11-14

**Authors:** Shraman Kadapa, Nicholas Marcouiller, Anthony C. Drago, James L. Tangorra, Harry G. Kwatny

**Affiliations:** Mechanical Engineering and Mechanics, Drexel University, Philadelphia, PA 19104, USA; nm875@drexel.edu (N.M.); ad892@drexel.edu (A.C.D.); jlt66@drexel.edu (J.L.T.); hgk22@drexel.edu (H.G.K.)

**Keywords:** modeling, simulation, marine hydrodynamics, bio-inspired robotics, dynamics, computational fluid dynamics

## Abstract

There is a growing demand for underwater robots to support offshore tasks such as exploration, environmental monitoring, and critical underwater missions. To enhance the performance of these systems, researchers are increasingly turning to biological inspiration to develop robots that understand and adapt the swimming strategies of aquatic animals. Numerical modeling plays a critical role in evaluating and improving the performance of these complex, multi-body robotic systems. However, developing accurate models for multi-body robots that swim freely in three dimensions remains a significant challenge. This study presents the development and validation of a numerical model of a bio-inspired California sea lion (*Zalophus californianus*) robot. The model was developed to simulate, analyze, and visualize the robot’s body motions in water. The equations of motion were derived in closed form using the Euler–Poincaré formulation, offering advantages for control and stability analysis. Hydrodynamic coefficients essential for estimating fluid forces were computed using computational fluid dynamics (CFD) and strip theory and further refined using a genetic algorithm to reduce the sim-to-real gap. The model demonstrated strong agreement with experiments, accurately predicting the translation and orientation of the robot. This framework provides a validated foundation for simulation, control, and optimization of bio-inspired multi-body systems.

## 1. Introduction

There has been a significant rise in the development of underwater robots, with the goal of improving the ability of these robots to swim and maneuver effectively in dynamic, high-energy flow environments [1,2,3]. This growth is driven by the increasing demand for robotic systems to assist with offshore tasks such as scientific research, critical underwater missions, and ocean exploration, needs recognized by organizations like the Office of Naval Research (ONR) and the National Oceanic and Atmospheric Administration (NOAA) [1,2,4,5]. These applications often require underwater robots to navigate tight spaces, avoid obstacles, operate at high speeds, and execute complex maneuvers with agility in energetic flows and occluded environments. To operate effectively in these conditions, underwater robots need to swim with great agility and maneuverability to take advantage of and adapt to multi-directional flows.

One promising approach to improve the performance of underwater robots is to develop robots that are able to understand and adapt the locomotion strategies of biological swimmers [6,7,8]. Many aquatic animals utilize their bodies and propulsors to swim and maneuver effectively in complex, unsteady flow environments. As a result, engineers want to adapt swimming techniques employed by biological systems onto underwater robots to improve swimming performance [9,10]. Current biologically inspired underwater robots often feature multiple body segments, fins, or flippers of various shapes, sizes, and degrees of freedom (DoFs). For example, fish robots utilize multiple fins to produce and shape propulsive forces [11,12], sea turtle robots use soft morphing flippers to swim and traverse on land [13,14], and sea snakes and eel robots have multiple articulated bodies [15,16,17] to navigate confined places. As these robotic platforms become more advanced, it is increasingly important to understand how the actuation of various body segments influences overall swimming and maneuvering performance.

Numerical modeling is a powerful tool for studying the biomechanics of multi-body underwater robots; however, existing approaches often rely on simplifying the model, which limits their applicability for complex bio-inspired systems. The limited multi-body models that do exist primarily focus on snake-like robots composed of uniform, symmetric links [18,19,20]. Although deriving the equations of motion in closed-form solutions provides significant advantages for stability analysis [21] and control design [18,20,22], such formulations have only been demonstrated for planar or similar linked systems rather than for freely swimming three-dimensional platforms with non-uniform segments. Unlike traditional underwater robots that rely on propellers for propulsion [23,24], bio-inspired unmanned underwater vehicles (UUVs) utilize propulsors such as fins and flippers that generate unsteady, three-dimensional, time-dependent forces that vary in direction throughout each stroke cycle [25]. Moreover, each articulated body segment is not necessarily uniform in shape, size, and mass. These differences affect the distribution of inertia, mass, and hydrodynamic forces for the entire system. This makes modeling particularly difficult, as each segment’s motion not only influences the forces and torques acting on itself but also affects adjacent segments, leading to strong interdependencies between the degrees of freedom. Another major challenge lies in the analytical computation of the hydrodynamic forces, which depends on accurate approximations of hydrodynamic coefficients [26]. Experimental methods, such as tow-tank experiments, have proven to be useful for computing these coefficients [27,28,29]. However, these methods require large, dedicated facilities, making them costly and often impractical. To address these challenges, a numerical modeling framework that integrates a closed-form dynamical model, accurately estimates hydrodynamic forces produced by multiple body segments, and provides effective kinematic visualization is essential.

**Figure 1:**
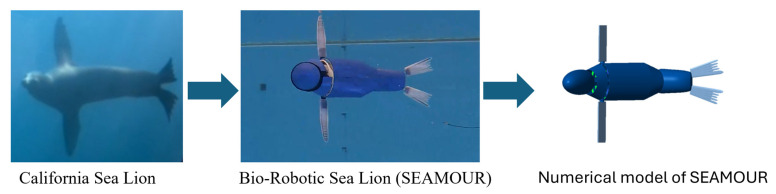
From biological system to engineered platform: California sea lion (**left**), bio-robotic sea lion (SEAMOUR—**center**), and numerical model (**right**).

To that end, the objective of this work was to develop and validate a numerical model for a biologically inspired sea lion robot using the Euler–Poincare formulation to simulate, analyze, and visualize the body motions of the robot in water (Figure 1). A bio-robotic sea lion was chosen for this study because its complex, multi-segmented body and three-dimensional flipper movements provided an ideal test case for validating the proposed methodology for the development of the numerical model. Additionally, as the robot was developed in-house, the authors had full access to its geometric and inertial properties, which were crucial for accurate model development and validation. A detailed description of the development of the bio-robotic platform can be found in [30]. While previous numerical models have addressed simplified or planar systems, this study, to the best of the authors’ knowledge, is the first to derive the equations of motion in a closed-form solution for a freely swimming, multi-body unmanned underwater vehicle (UUV) with non-uniform body segments. The critical difference between our approach and existing models lies in our framework, which overcomes the inherent challenges of modeling such systems. The equations of motion for the entire model were solved symbolically using the Euler–Poincare formulation [22], which takes the generalized form of Lagrange’s equations. Unlike Lagrange’s equations of motion that rely on the time derivative of the generalized coordinates, the Euler–Poincare model uses quasi-velocities, which are velocity components defined in the local body-fixed frame [22]. This formulation made it easier to estimate the hydrodynamic forces produced by the articulation of different body segments. To accurately compute these forces, we developed a hybrid approach combining computational fluid dynamics (CFD) with analytical strip theory. Another significant contribution to this work is the application of a genetic algorithm to optimize hydrodynamic coefficients and other key parameters, which ensured a close alignment between our numerical model and the physical robot’s performance. This optimization step is vital; it addresses a major challenge in aquatic robotics by effectively reducing the sim-to-real gap and providing an accurate, predictive tool for motion analysis and control. To visualize the kinematics of the vehicle, the Euler–Poincare model was integrated with MATLAB Simscape (The MathWorks, Inc., MATLAB version: 9.14.0 (R2023a) Update 1, Portola Valley, CA, USA) to show how the bio-robotic platform moves over time. The joints and their respective kinematics in the numerical model were controlled using trajectory tracking controllers. These controllers were developed using extensions of feedback linearization methods to accurately follow the desired motion path.

Numerical modeling has proven effective to analyze the performance of multi-body robotic systems in terrestrial and aerial domains; however, its application in the underwater domain remains limited due to the challenges of capturing the dynamics of multi-body articulation of non-uniform body segments in a fluid environment. In the terrestrial domain, dynamic models of legged robots have been developed with accurate representations of ground contact forces, which enabled precise control and analysis of multi-limbed locomotion [31,32,33]. In the aerial domain, numerical models have been developed to capture the dynamics of both biologically inspired and engineered systems. For example, a numerical model of the common guillemot was developed to capture the kinematic and inertial effects of its flapping wings during aerial locomotion [34] and demonstrated how articulated control surfaces could be effectively modeled. Similarly, ref. [35] developed a numerical model of a bird-scale aerial vehicle that incorporated the effects of inertia change in wings and their passive deformation effects to analyze and demonstrate flight stability. In the underwater domain, early research primarily focused on torpedo and box-shaped vehicles, with studies such as [23,24] using classical numerical modeling techniques based on the [26] approach. Building on these foundations, only a few numerical models have been developed for bio-inspired underwater robots. One such study [36] modeled an octopus-inspired robot capable of underwater legged locomotion (ULL). This work uniquely combined hydrodynamic sculling forces with ground contact forces to simulate benthic propulsion, demonstrating how body morphology, buoyancy distribution, and multi-modal actuation can influence underwater locomotion in shallow or near-bottom environments. Most existing numerical models for multi-body underwater systems have been developed for snake-like robots, which possess uniform body segments using both closed-form and non-closed-form approaches [18,37,38,39]. Another important aspect of underwater robot modeling lies in accurately estimating the hydrodynamic forces. While many have computed coefficients using tow-tank experiments, more recently, computational fluid dynamics (CFD) methods [24,40,41] have been increasingly used, demonstrating effectiveness and offering results comparable to experimental approaches. Thus, there remains a clear need for a physics-based, closed-form numerical framework capable of accurately capturing the hydrodynamics and kinematics of a freely swimming, multi-segmented, non-uniform underwater vehicle.

The remainder of this paper will discuss the following: (1) derivation of the closed-form dynamical equations of motion for a bio-robotic sea lion platform using the Euler–Poincare formulation; (2) determination of hydrodynamic coefficients using CFD and strip theory; and (3) experimental results of the bio-robotic sea lion executing flipper and body motions with numerical model validation.

## 2. Materials and Methods

### 2.1. Overview

A numerical model was developed to simulate, visualize, and analyze the body kinematics for a biologically inspired sea lion robot during different swimming and maneuvering trials. The equations of motion for the bio-robotic swimmer were derived in closed form using the Euler–Poincare approach [22] (Section 2.2). To simulate the articulation of individual body segments in the UUV, independent trajectory tracking controllers were developed that followed the prescribed kinematics using extensions of feedback linearization methods [22] (Section 2.3). Hydrodynamic forces that were produced by the articulation of various body segments of the robotic swimmer were analytically computed and applied to the center of mass of each body segment in the numerical model (Section 2.4). The hydrodynamic coefficients, which were essential for hydrodynamic forces, were computed using computational fluid dynamics simulations and strip theory. A genetic algorithm was then used to refine these coefficients, which reduced the sim-to-real gap in terms of performance between the robotic swimmer and its numerical model. For visualization of the kinematics of the numerical model, the Euler–Poincare model was integrated into the MATLAB Simscape environment (Section 2.5). Performance metrics were then defined that allowed comparison and validation of the numerical model against the experimental platform (Section 2.6).

**Figure 2:**
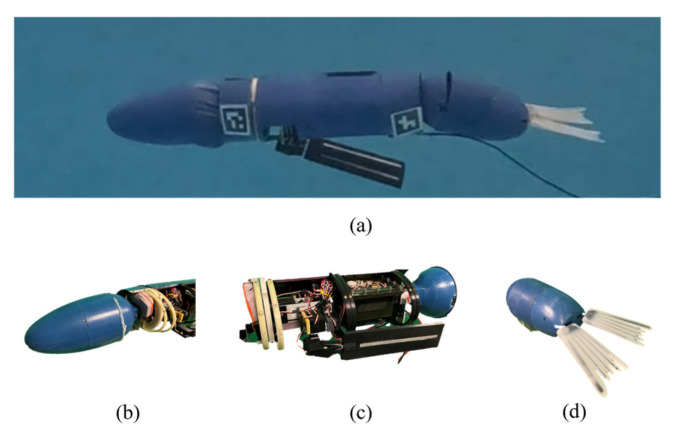
Bio-robotic sea lion with key anatomical sections. (**a**) Complete assembly, (**b**) head, (**c**) main body section with fore flippers, and (**d**) pelvis section with hind flippers.

**Figure 3:**
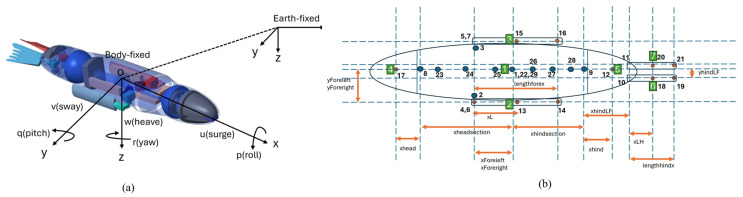
Reference frame and the mechanical tree (illustration) for the numerical model. (**a**) Body fixed frame for the bio-robotic sea lion. (**b**) Mechanical tree with outboard nodes showcasing the rigid connection between various control surfaces, such as the head at node 4, pelvis at node 5, a pair of fore at nodes 2 and 3, and hind flippers at nodes 6 and 7.

The bio-robotic sea lion, referred to as the Stroke Experimentation and Maneuver Optimizing Underwater Robot (SEAMOUR) (Figure 2a), was developed to investigate how sea lions utilize their flippers and body to swim and maneuver. The robotic system comprises seven major body segments: a main body, head (Figure 2b), a pair of fore flippers (Figure 2c), pelvis (Figure 2d), and a pair of hind flippers. The head and pelvis sections, each with two degrees of freedom in pitch and yaw relative to its body fixed frame (Figure 3a), were both connected to the central main body. A pair of rigid fore flippers was attached to the main body with three degrees of freedom (roll, pitch, and yaw) each, while a pair of rigid hind flippers (Figure 2d) was connected to the pelvis with two degrees of freedom (roll and yaw). SEAMOUR is an open system that allows for water ingress and egress during operation. An external antenna was mounted on a small surface float during experiments. SEAMOUR is set to be neutrally buoyant with horizontally level orientation. A comprehensive description of SEAMOUR’s design, fabrication, and component specifications has previously been described in [30,42].

### 2.2. Derivation of Closed-Form Equations of Motion (EoM)

**Figure 4:**
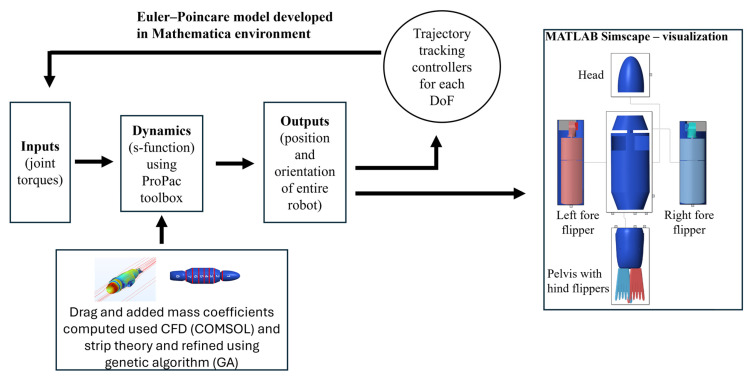
Numerical model layout—equations of motion solved in the Poincare model and the Simscape environment used for visualization.

The numerical model of the bio-robotic sea lion was developed using the Euler–Poincare formulation, which takes the generalized form of the Euler–Lagrange equations [22]. In this approach, the equations of motion are expressed using the quasi-velocity variables, which define the linear and angular velocities in the local body-fixed frame (Figure 3a). This differs from the traditional Lagrangian method, where velocities are defined as the time derivatives of the generalized coordinates [22]. The equations of motion were symbolically derived in Mathematica (Wolfram Research, Inc.,Wolfram Mathematica 14, Champaign, IL, USA (2024)) using ProPac [22], a toolbox that integrates into Mathematica as an add-on package. ProPac provides the necessary kinematic and dynamic functions for modeling multi-body vehicles. A schematic of the overall numerical model structure is shown in Figure 4.

SEAMOUR was modeled as a mechanical tree structure, where the system is represented as a chain of interconnected bodies [22]. Each body contained a distinct node, which served either as a connection point to another body segment or as the location where generalized forces were applied. The node placements for the various body segments of SEAMOUR are illustrated in Figure 3b. Figure 3b is provided for illustration of the mechanical tree structure; the geometric, mass, and inertial properties used in the numerical model are derived directly from the bio-robotic sea lion. The geometrical features of the numerical model, including segment dimensions and inter-segment distances, are provided in Supplementary Material File S1 for reference. The main rigid body had 6 degrees of freedom (3 translation and 3 rotation). The head and pelvis were both connected to the main body with 2 degrees of freedom. A pair of hind flippers was connected to the pelvis with 2 degrees of freedom each. In total, the model encompassed 6 degrees of freedom (DoF) in the Cartesian space and 14 degrees of freedom in the individual joint space. To simulate the kinematics of these body segments, individual trajectory tracking controllers were developed; further details are provided in Section 2.3. The position and orientation variables were defined using the SNAME notation [40]. The generalized coordinates associated with this entire model are represented by a vector q of length 20 composed of the following elements:

(1) $$q_{main\;body}={\left[\varphi_{1},\theta_{1},\psi_{1},x,y,z\right]}^{T},$$

(2) $$q_{foreflipper}={\left[\varphi_{i},\theta_{i},\psi_{i}\right]}^{T}$$

where *i* = 2, 3, representing the left and right fore flippers,

(3) $$q_{hindflipper}={\left[\varphi_{i},\psi_{i}\right]}^{T}$$

where *i* = 4, 5, representing the left and right hind flippers,

(4) $$q_{head}={\left[\theta_{6},\psi_{6}\right]}^{T},$$

(5) $$q_{pelvis}={\left[\theta_{7},\psi_{7}\right]}^{T},$$

where φ,θ,ψ (*i* = 1, 2,…,7) represents the rotations about X, Y, and Z axes for each body part. Each vector q specifies a configuration of 7 bodies. The set of all configurations is a smooth manifold called the configuration space, *M*. At each point q∈M, the time derivative of q is the generalized velocity $\dot{q}$.

The time derivative of the generalized coordinates, i.e., the generalized velocity, $\dot{q}$, is related to the quasi-velocity, p, using the following equations:

(6) $$p_{main\;body}={\left[p_{1},q_{1},r_{1},u,v,w\right]}^{T},$$

(7) $$p_{foreflipper}={\left[p_{i},q_{i},r_{i}\right]}^{T}$$

where *i* = 2, 3, representing the left and right fore flippers,

(8) $$p_{hindflipper}={\left[p_{i},q_{i},r_{i}\right]}^{T}$$

where *i* = 4, 5, representing the left and right hind flippers,

(9) $$p_{head}={\left[q_{6},r_{6}\right]}^{T},$$

(10) $$p_{pelvis}={\left[q_{7},r_{7}\right]}^{T}.$$

The notation $p_{i},q_{i},r_{i}$ (*i* = 1, 2,…, 7) represents the time angular velocities about the joint X, Y, and Z axes for each body part. The kinematics of this multi-body model are represented by Equation (11):

(11) $$\dot{q}=V\left(q\right)p.$$

(12) $$q=\left[\begin{array}{c} q_{main\;body};q_{leftfore};q_{rightfore};\\ q_{lefthind};q_{righthind};q_{head};q_{pelvis} \end{array}\right],$$

(13) $$p=\left[\begin{array}{c} p_{main\;body};p_{leftfore};p_{rightfore};\\ p_{lefthind};p_{righthind};p_{head};p_{pelvis} \end{array}\right]$$

where *q* is the generalized coordinates vector and *p* is the quasi-velocity vector, each of length 20. The matrix $V\left(q\right)$ is the velocity transformation matrix, a 20 × 20 square matrix of full rank. The columns of $V\left(q\right)$, $v_{i}\left(q\right),i=1,\ldots ,\;20$ form a set of independent vector fields on the configuration manifold *M.* The construction of these vector fields is based on joint configurations.

Similar to the standard form of dynamical equations used for underwater vehicles [26], the dynamics (Figure 4) of SEAMOUR are represented by Equation (14):

(14) $$M\left(q\right)\dot{p}+C\left(p,q\right)p+F\left(p,q\right)=Q$$

where *M(q)* is the mass matrix (20 × 20), the matrix *C*(*p*,*q*) contains centripetal and Coriolis components (20 × 20), *F*(*p*,*q*) is the force vector internal to the system (20 × 1), and *Q* is the vector of externally applied generalized forces (20 × 1). The mass and inertial properties for various body segments are presented in Table 1. The mass and Coriolis matrices have a full rank of 20, which includes all degrees of freedom and body segments. Figure 3b shows all variables considered to describe the geometrical features of the model. To represent the full mass matrix, these matrices were decomposed into submatrices (15–19). To simplify the complete set of equations, the system is in a neutral configuration, where the fore flippers remain streamlined next to the main body. Additionally, to simplify the equations here, it is assumed that all body segments, such as the head, pelvis with hind flippers, fore flippers, and main body, are not moving, and their respective linear and angular velocities are zero.

(15) $$M=\left[\begin{array}{cc} M11_{6x6} & M12_{6x14}\\ M21_{14x6} & M22_{14x14} \end{array}\right]$$

(16) $$M11_{6x6}=\left[\begin{array}{cccccc} 0.13 & 0 & 0.001 & 0 & 0.347 & 0\\ 0 & 1.64 & 0 & -0.347 & 0 & 0.68\\ 0.001 & 0 & 1.68 & 0 & -0.68 & 0\\ 0 & -0.347 & 0 & 16.1 & 0 & 0\\ 0.347 & 0 & -0.68 & 0 & 16.1 & 0\\ 0 & 0.68 & 0 & 0 & 0 & 16.1 \end{array}\right]$$

(17) $$M12_{6x14}=\;\left[\begin{array}{cccccccccccccc} 0.006 & 0.006 & 0.001 & 0.001 & 9e-5 & 9e-5 & 0 & 0 & 0 & 0 & 7e-5 & 0 & 7e-5 & 0\\ -0.0004 & 0.0004 & \;0 & 0 & 0 & 0 & 0.12 & 0 & 0.21 & 0 & 0 & 0 & 0 & 0\\ 0.0006 & 0.0006 & 0.0007 & 0.0007 & 0 & 0 & 0 & 0.12 & 0 & 0.21 & 0 & 0.008 & 0 & 0.008\\ 0 & 0 & 0 & 0 & 0 & 0 & 0 & 0 & 0 & 0 & 0 & 0 & 0 & 0\\ -0.006 & -0.006 & -0.04 & -0.04 & 0 & 0 & 0 & 0.33 & 0 & -0.37 & 0 & -0.013 & 0 & -0.013\\ -0.004 & 0.004 & 0 & 0 & 0 & 0 & -0.33 & 0 & 0.37 & 0 & 0 & 0 & 0 & 0 \end{array}\right]$$

(18) $$M21_{14x6}=\left[\begin{array}{cccccc} 0.006 & -0.0004 & 0.0005 & 0 & -0.006 & -0.004\\ 0.006 & \;0.0004 & 0.0005 & 0 & -0.006 & 0.004\\ 0.001 & 0 & 0.0007 & 0 & -0.04 & 0\\ 0.001 & 0 & 0.0007 & 0 & -0.04 & 0\\ 9e-5 & 0 & 0 & 0 & 0 & 0\\ 9e-5 & 0 & 0 & 0 & 0 & 0\\ 0 & 0.12 & 0 & 0 & 0 & -0.33\\ 0 & 0 & 0.12 & 0 & 0.33 & 0\\ 0 & 0.21 & 0 & 0 & 0 & 0.37\\ 0 & 0 & 0.21 & 0 & -0.37 & 0\\ 7e-5 & 0 & 0 & 0 & 0 & 0\\ 0 & 0 & 0.008 & 0 & -0.013 & 0\\ 7e-5 & 0 & 0 & 0 & 0 & 0\\ 0 & 0 & 0.008 & 0 & -0.013 & 0 \end{array}\right]$$

(19) $${M22}_{14x14}=\;\left[\begin{array}{cccccccccccccc} 0.006 & 0 & 0.001 & 0 & 9e-5 & 0 & 0 & 0 & 0 & 0 & 0 & 0 & 0 & 0\\ 0 & 0.006 & 0\; & 0.001 & 0 & 9e-5 & 0 & 0 & 0 & 0 & 0 & 0 & 0 & 0\\ 0.001 & 0 & 0.005 & 0 & 0 & 0 & 0 & 0 & 0 & 0 & 0 & 0 & 0 & 0\\ 0 & 0.001 & 0 & 0.005 & 0 & 0 & 0 & 0 & 0 & 0 & 0 & 0 & 0 & 0\\ 9e-5 & 0 & 0 & 0 & 9e-5 & 0 & 0 & 0 & 0 & 0 & 0 & 0 & 0 & 0\\ 0 & 9e-5 & 0 & 0 & 0 & 9e-5 & 0 & 0 & 0 & 0 & 0 & 0 & 0 & 0\\ 0 & 0 & 0 & 0 & 0 & 0 & 0.045 & 0 & 0 & 0 & 0 & 0 & 0 & 0\\ 0 & 0 & 0 & 0 & 0 & 0 & 0 & 0.045 & 0 & 0 & 0 & 0 & 0 & 0\\ 0 & 0 & 0 & 0 & 0 & 0 & 0 & 0 & 0.06 & 0 & 0 & 0 & 0 & 0\\ 0 & 0 & 0 & 0 & 0 & 0 & 0 & 0 & 0 & 0.06 & 0 & 0.003 & 0 & 0.003\\ 0 & 0 & 0 & 0 & 0 & 0 & 0 & 0 & 0 & 0 & 7e-5 & 0 & 0 & 0\\ 0 & 0 & 0 & 0 & 0 & 0 & 0 & 0 & 0 & 0.003 & 0 & 0.0004 & 0 & 0\\ 0 & 0 & 0 & 0 & 0 & 0 & 0 & 0 & 0 & 0 & 0 & 0 & 7e-5 & 0\\ 0 & 0 & 0 & 0 & 0 & 0 & 0 & 0 & 0 & 0.003 & 0 & 0 & 0 & 7e-5 \end{array}\right]$$

It is important to note that the above full mass matrix of rank 20 is symmetric. Additionally, the Coriolis and centripetal matrix typically depend on the linear and angular velocities of the generalized coordinates. Since we have assumed these coordinates are zero in the above displayed equations of motion, the centripetal matrix is zero. Full ranked mass, Coriolis and centripetal, and force matrices can be seen in the Mathematica file, which is available to the reader upon reasonable request to the authors. To better understand how this modeling approach was used to generate equations of motion for this multi-body system, please refer to [22].

The mass matrix in symbolic form for the entire model is provided in Supplementary Material File S1. The principal and product moments of inertia were calculated using a computer-aided design (CAD) model of SEAMOUR on CREO [Creo Parametric, PTC, Boston, MA, USA]. In addition to these inertial parameters, the total mass of the main body was adjusted to include the additional water mass from the UUV’s flooded section. The CAD model was used to determine the geometrical properties of the main body and its various segments, while each body segment was individually weighed to determine its dry mass. The mass and geometric parameters used in the numerical model of SEAMOUR are summarized in Table 1.

### 2.3. Trajectory Tracking Controllers

**Figure 5:**
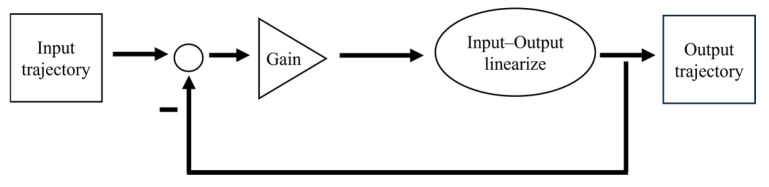
General architecture for individual trajectory tracking controllers.

Individual tracking controllers were developed to control the fore and hind flippers, head, and pelvis with desired trajectories (Figure 5). A two-level strategy was used that first implemented the feedback linearizing control and then linear feedback to regulate the linearized system [22]. For each degree of freedom (roll, pitch, and yaw) in the fore flippers, individual trajectory tracking controllers were developed. For each of the head, pelvis, and hind flippers, a 2-DoF tracking controller was developed. A detailed description of the model setup and the use of the ProPac toolbox is described in [22].

### 2.4. Hydrodynamic Forces

Hydrodynamic forces such as drag, lift, lateral, and added mass forces were incorporated into the numerical model to simulate SEAMOUR swimming and maneuvering in water. As shown in (14), which defines the equations of motion, vector Q represents the total external hydrodynamic forces acting on the system. These forces include the effects of drag, lift, and lateral forces, applied to each body segment. Drag forces in the main body were modeled as resistive forces acting opposite to the *X* axis (axial direction), as shown in Figure 3a. Lateral forces are applied along the *Y* axis, and lift forces are applied along the *Z* axis. Using the Generalized Force function within the ProPac toolbox, hydrodynamic forces were computed and applied at the center of mass nodes for the fore flippers, hind flippers, head, and pelvis. For the main body, drag and added mass forces in the X direction were applied to its center of mass. To account for changes to relative velocities across the body in Y and Z directions, the entire model was subdivided into eight segments, including the head, pelvis, and six sections along the main body. Forces were applied to the center of mass of each segment and scaled according to their proportional surface area in the relevant direction. The force coefficients for drag, lift, and lateral components were obtained through computational fluid dynamics (CFD) simulations, while the added mass coefficients were computed analytically using strip theory, applied individually to each body segment.

Some assumptions were taken to simplify the computation of the hydrodynamic coefficients. The main body was considered a prolate spheroid, and the fore flippers were considered rectangular flat plates. Water was assumed to be inviscid, incompressible, and irrotational for the computation of added mass coefficients—an assumption commonly adopted in prior studies, such as in [26]. It was also assumed that the model has three planes of symmetry, with the model always completely submerged in water. In addition, the center of mass and center of buoyancy for each flipper, as well as the head and pelvis sections, are coincident. All body segments were assumed to be individually neutrally buoyant.

**Figure 6:**
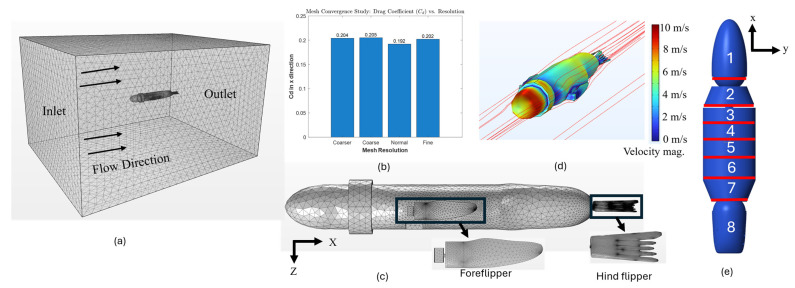
Coefficients of force computed for the entire model in all axes using computational fluid dynamics simulations. (**a**) Mesh for fluid domain. (**b**) Mesh resolution vs. drag coefficient (x). (**c**) User-controlled mesh generated for the bio-robotic sea lion. (**d**) Velocity magnitude when computing the coefficient of drag for the robot. (**e**) Coefficient of drag computed along the y and z axes for each 1/8th segment of the full numerical model.

Computational fluid dynamics (CFD) simulations were developed to determine the force coefficients in all directions for each body segment of SEAMOUR (Figure 6). The CAD geometry of the main body was directly imported into COMSOL Multiphysics 6.3, where a user-controlled mesh was utilized, with the element size calibrated for fluid dynamics (Figure 6a,c). A fine mesh resolution was selected with free tetrahedral elements. The calculated force coefficients were insensitive to mesh resolution (Figure 6b). The maximum and minimum element sizes were 0.184m and 0.0346m, respectively. The maximum element growth rate was 1.13 with a curvature factor of 0.5, and the resolution of narrow regions was set at 0.8. The complete mesh contained 4.04 × 10^6^ domain elements. The entire computational domain was 5 m × 4 m × 3 m (Figure 6a). A k–ε turbulence model was used, with an inflow velocity of 10 m/s applied across the model domain (Re ~$9\times 10^{6}$). No slip boundary condition was used for the walls. To calculate the force coefficients, the line integral of the total stress along the boundaries of each geometry was evaluated, yielding force coefficients along the X, Y, and Z axes. For the X-direction force coefficient of the main body (Figure 6d), the full CAD assembly was imported into COMSOL. For force coefficients in the y and z directions, each body segment was imported individually (Figure 6e), and CFD simulations were run separately to obtain the segment-specific coefficients. Similarly, for both the fore flippers and hind flippers, each flipper’s geometry was analyzed independently to compute force coefficients in all three directions. Drag forces acting on the main body, head, pelvis, and flippers were calculated using (20). Additionally, the velocities at the center of mass nodes were sensed and subsequently used to compute forces at the center of mass nodes of each flipper as well as the body segment. The *NodeVelocity* function in ProPac was used to sense the velocities at every node of the numerical model [22]. More information on this can be found in the attached Supplementary Materials. The drag force equation is

(20) $$F_{D}=\frac{C_{D}\rho Av\left|v\right|}{2}.$$

where F_D_ is the drag force exerted, C_D_ is the coefficient of drag, ρ is the density of water, A is the reference area, and v is the velocity of the object relative to the fluid.

To compute the added mass forces, it was important to compute the added mass coefficients, which are used in (27) [26]. Due to the symmetrical shape of the model, the added mass coefficients related to the product of two axes were ignored. To calculate these coefficients for the main body, ref. [37] provided formulas that utilize the geometrical properties of the body. The eccentricity of the prolate spheroid and the added mass coefficients for a prolate spheroid are given below:

(21) $$e=\sqrt{1-{\left(\frac{b}{a}\right)}^{2}},$$

(22) $$\alpha_{0}=\frac{2\left(1-e^{2}\right)}{e^{3}}\left(\frac{1}{2}ln\frac{1+e}{1-e}-e\right),$$

(23) $$\beta_{0}=\frac{1}{e^{2}}-\frac{1-e^{2}}{2e^{3}}ln\frac{1+e}{1-e}.$$

(24) $$X_{\dot{u}}=-\frac{\alpha_{0}}{2-\alpha_{0}}m,$$

(25) $$Y_{\dot{v}}=Z_{\dot{w}}=-\frac{\beta_{0}}{2-\beta_{0}}m.$$

To compute the hydrodynamic added mass coefficients for the fore and hind flippers, strip theory was employed. This approach involved dividing the submerged shape into strips, computing the two-dimensional cross-sectional coefficients, and integrating them along the shape’s length to determine the three-dimensional coefficients (Figure 6c). These added mass coefficients were computed based on methodologies presented by [43], and (26) was used:

(26) $$M_{a}=\;k_{trans}\frac{\pi \rho c^{2}b}{4}.$$

where K_trans_ is the coefficient of additional mass as a function of aspect ratio of rectangular flat plate in Figure 3 [31], where ρ is the density of water, c is the chord, and b is the span.

(27) $$Added\;mass=M_{a}.a$$

where a is the acceleration sensed at the same node. In the Simscape multibody environment, a transform sensor block was placed between the body frame and the world frame to sense accelerations in all axes at various nodes. Coefficients of force for the main body as well as the front face of the fore flippers are presented in Table 1.

A genetic algorithm was used to obtain more accurate estimates of the hydrodynamic coefficients and the volume of water ingress within SEAMOUR. The calculation of hydrodynamic derivatives using empirical formulas is particularly challenging for robots with complex shapes, as existing formulas in the literature are typically based on simplified geometries. The robot’s main body was initially approximated as a prolate spheroid with equal minor axes to simplify the computation of added mass coefficients. The cross-sectional profile of the body was elongated along the *Y*-axis relative to the *Z*-axis, introducing asymmetries. In addition, because SEAMOUR was built for water going in, the internal water volume was treated as a variable in the optimization process. Analytical modeling of water ingress and egress in SEAMOUR poses a significant challenge, as the amount of water entering the system varies with the robot’s speed and orientation. The optimized parameters included the volume of water inside SEAMOUR, the added mass coefficient in the z direction, the buoyancy offset in the main body, and the drag coefficient in the z direction. To address these challenges, an optimization process focused on minimizing an error function based on Dynamic Time Warping (DTW) using a multi-objective approach was used. The two primary objectives were ensuring accurate alignment between the robot’s position and the numerical model, as well as a consistent pitch angle between the model and the robot for the complete duration during different strokes. The algorithm was configured with a population size of 80 and with a maximum generation of 50, with a crossover fraction of 0.8; the Pareto function was 0.4. The genetic algorithm was applied to two distinct trajectories: (1) the characteristic stroke, a gait commonly observed in sea lions, and (2) the characteristic stroke with an actuated pelvis. Following the optimization process, the resulting coefficients were fine-tuned manually to identify values that worked best for both trajectories. These refined parameters were then used for the analysis of swimming and maneuvering tests.

### 2.5. Simscape Visualization

A multibody assembly of the bio-robotic sea lion was developed using MATLAB’s Simscape environment to visualize the 6-DoF body movements (Figure 4). This assembly served as a dynamic representation of the robot’s kinematics. Each anatomical section of the bio-robotic sea lion—such as the main body, head, pelvis, fore and hind flippers—was modeled in CREO and imported into the Simscape environment. Each body segment was connected to the main body with revolute joints equivalent to its degrees of freedom. The main body of the model was connected to the simulation environment through a 6-DoF joint to visualize translation and rotation of the entire assembly. Outputs from the Poincare model were directly integrated into the Simscape model for visualization.

### 2.6. Model Validation Experiments and Data Processing

Experiments were conducted in a pool to validate the numerical model against SEAMOUR. First, to ensure that various body segments in the numerical model followed the prescribed trajectories, the performance of the individual trajectory tracking controllers was evaluated by analyzing the differences between the input and output trajectories. Following this, a characteristic stroke of a sea lion, as previously described in [28], was implemented on both SEAMOUR and the numerical model. The same stroke was then tested with additional pelvis actuation to compare the response between the two systems. Lastly, simultaneous actuation of the head and pelvis sections in both pitch and yaw was conducted to validate the numerical model against SEAMOUR during maneuvers.

To quantify the kinematic validation of the center of mass of the numerical model against the robot, several metrics were defined. These included the following: total distance traveled in surge and heave directions, maximum and mean resultant velocity magnitude for every cycle within a stroke, final pitch angle after every cycle, and maximum and mean angular velocity during each cycle of a stroke. For the maneuver tests, final orientation as well as maximum and mean angular velocity at the end of four seconds were recorded. For the evaluation of the performance of the tracking controller, rise time and settling time are used to describe the differences between the input and output trajectories.

**Figure 7:**
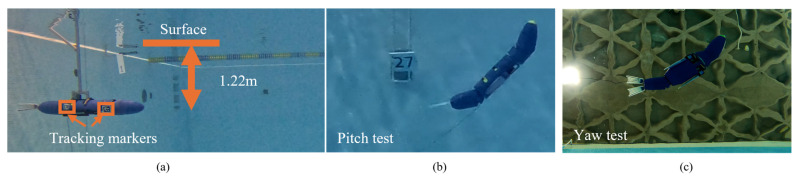
Experimental setup for robotic system on the dock for swimming and maneuvering trials. (**a**) Bio-robotic sea lion magnetically docked to the aluminum structure for a consistent initial position at approximately 1.22m depth. (**b**) Actuated head and pelvis for pitch maneuver. (**c**) Actuated head and pelvis for yaw maneuver with a camera on the bottom of the pool.

SEAMOUR was tested in a pool measuring 9 m × 9 m × 4 m (length × width × depth), where videos were recorded to capture its position and orientation across different trials. To ensure a consistent starting position and orientation, the robot was magnetically attached at its midsection to an aluminum docking structure mounted against one wall of the pool. The docking system included a magnetic end effector with prongs to secure the robot prior to each trial. All experiments were conducted at a depth of approximately 1.22 m. Video footage was captured using a GoPro Hero 13 (GoPro, San Mateo, CA, USA) mounted on the adjacent pool wall, recording at 30 frames per second in 4K resolution. A reference scale with a known length was used in the field of view to convert pixel measurements to meters. Processed clips were then imported into MATLAB, where the Image Processing and Computer Vision Toolboxes were used to track the robot’s motion. Two visual markers (Figure 7a), placed on the head and pelvis shells, were used to extract position and pitch angle data over the duration of each trial. During maneuver tests, orientation data were also recorded by an onboard inertial measurement unit (BNO085, PN 4754, Adafruit, Brooklyn, NY, USA), at a sampling rate of 20 Hz. Each maneuver trial began with three paddle strokes to bring SEAMOUR up to speed, followed by simultaneous actuation of the head and pelvis sections. The fore flippers were kept in a streamlined configuration and were not actuated during maneuvers. Although the fore flippers used for these tests were flexible and similar to those described in [42], they were treated as rigid for the purposes of this analysis. Since they were not actuated during the trials, their influence on overall performance was considered negligible. Separate video recordings were made for different types of maneuvers. For pitch trials (Figure 7b), the camera was mounted on the side wall, and for yaw trials (Figure 7c), a camera was placed on the bottom of the pool. To ensure fair comparison with numerical simulations, each simulation was initialized with the same pre-actuation velocity measured from experiments (approximately 0.21 m/s). Three trials were conducted for each test condition, and the results were averaged to quantify the robot’s position and orientation.

The numerical model was solved within the MATLAB Simscape environment. A variable-step ODE45 (Dormand-Prince) solver was used to solve the differential equations. The maximum step size was taken to be 0.1 with an automatically determined minimum step size depending on the trial. The relative tolerance was set at 1 × 10^−3^.

## 3. Results

### 3.1. Effectiveness of Trajectory Tracking Controller

**Figure 8:**
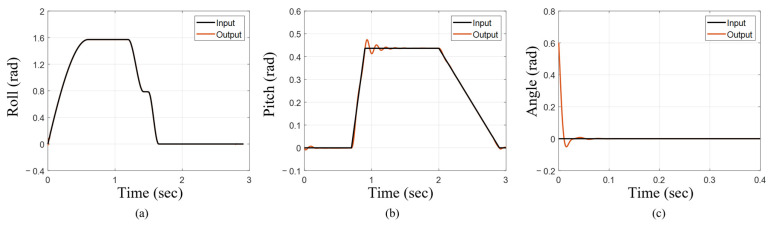
Effectiveness of individual trajectory tracking for various inputs. (**a**) Fore flipper roll during execution of characteristic sea lion stroke. (**b**) Pelvis pitch during free swimming test. (**c**) Non-zero initial condition for trajectory tracking.

Across different input trajectories, the tracking controllers consistently generated outputs that closely aligned with the desired input signals. The flipper roll trajectory was tracked near perfectly (Figure 8a). When the pelvis pitch trajectory was implemented, the tracking controller achieved a 2% settling time of 0.58 s (Figure 8b). Furthermore, for a steady-state input of zero with an initial condition of 0.6 radians, the controller exhibited a 2% settling time of just 0.017 s (Figure 8c), demonstrating strong agreement between input and output.

### 3.2. Linear Position and Velocity During Characteristic Stroke

**Figure 9:**
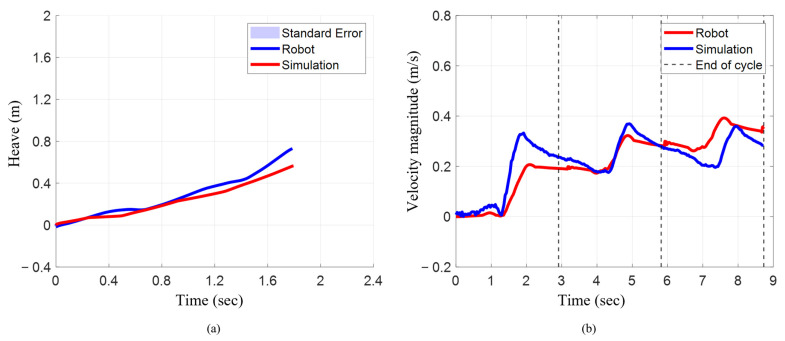
Position and resultant velocity magnitude when executing characteristic stroke. (**a**) Surge vs. heave for robot and simulation. (**b**) Resultant velocity magnitude.

The numerical model closely matched the bio-robotic system’s surge translation but exhibited a larger deviation in heave. When the characteristic stroke was implemented in the bio-robotic system, it translated 1.79 m in surge, while on the numerical model, it translated to 1.80 m, which was only 0.56% higher (Figure 9a). In heave, however, the robot translated 0.73 m, whereas the model reached 0.57 m (22% lower).

The mean resultant velocity magnitude increased progressively for each cycle for both the bio-robotic system and the numerical model (Figure 9b). In the numerical model, the mean velocity increased from 0.09 m/s in cycle 1 to 0.24 m/s (~167%) in cycle 2 and 0.32 m/s (~256%) in cycle 3. Similarly, the robot increased from 0.15 m/s in cycle 1 to 0.26 m/s (~73%) and 0.27 m/s (~80%) in cycles 2 and 3. Comparing both systems, the robot had a higher mean in cycle 1 (~67%), similar means in cycle 2 (~8% higher), and a slightly lower mean in cycle 3 (~16%).

The maximum resultant velocity magnitude increased for each cycle for the numerical model, but for the robotic system, it increased from cycle 1 to cycle 2 and slightly reduced in cycle 3. In the numerical model, it increased from 0.21 m/s to 0.32 m/s (52%) and 0.39 m/s (86%). For the robot, it rose from 0.33 m/s to 0.37 m/s (12%) and slightly decreased to 0.36 m/s (9%) (Table 2). While the robot exhibited higher maximum velocity in cycle 1 (57%), the values converged in later cycles, with only an 11% and 8% difference in cycles 2 and 3, respectively.

### 3.3. Angular Position and Angular Velocity During Characteristic Stroke

**Figure 10:**
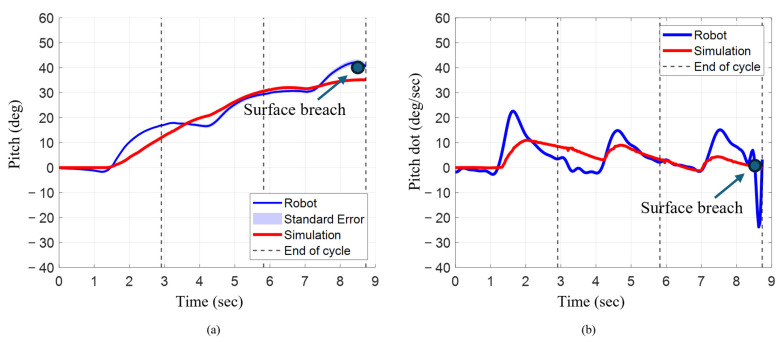
Orientation and angular rate for robot and simulation during characteristic stroke. (**a**) Pitch angle changes across three strokes. (**b**) Angular velocity across three strokes.

Final pitch angle increased progressively after each cycle for the characteristic stroke for both the bio-robotic system as well as the numerical model (Figure 10a). In the numerical model, pitch rose from 12° after cycle 1 to 31° (~158%) in cycle 2 and 35° (~11%) in cycle 3. For the robot, pitch increased from 17° to 29° (~71%) and 42° (~45%). The robot’s pitch was 42% higher after cycle 1, similar (~7% higher) after cycle 2, and 35% higher after cycle 3. 

The mean angular velocity followed an inconsistent trend between the numerical model and the robotic system across each cycle when the characteristic stroke was executed (Figure 10b). For the numerical model, it increased from 5°/s to 6°/s (20%) and decreased to 2°/s, while for the robot, it decreased from 6°/s to 4°/s (−33%) and then rose to 5°/s (+25%). When comparing both systems, in cycle 1, the mean was relatively similar, with the robot being 20% higher. In cycle 2, the mean angular velocity of the model was 50% higher. During the third cycle, the mean angular velocity of the robot was significantly higher than the numerical model, by almost 150%.

The maximum angular velocity for each cycle decreased for the numerical model, but for the robotic system, it decreased from cycle 1 to cycle 2 and slightly increased in cycle 3. For the numerical model, the maximum angular velocity for cycle 1 was approximately 11°/s. The max angular velocity decreased to approximately 9°/s (~18% lower). The max velocity further decreased by about 56% to 4°/s. For the robotic system, the max angular velocity in cycle 1 was 23°/s. The max angular velocity decreased in cycle 2 and cycle 3 to about 15°/s. For each cycle, the maximum angular velocity was consistently higher in the robot compared to the numerical simulation.

### 3.4. Linear Position and Velocity During Characteristic Stroke with Pelvis Actuation

**Figure 11:**
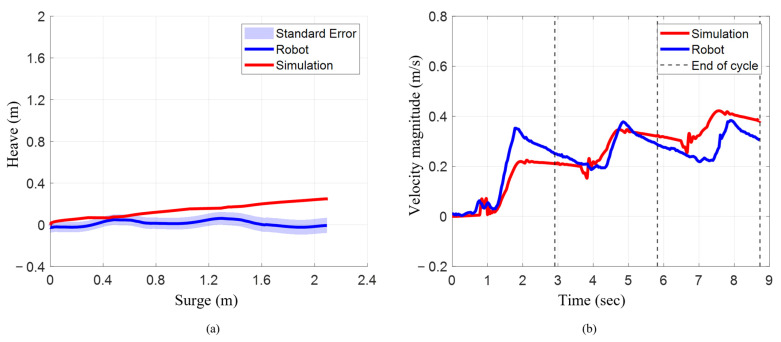
Position and resultant velocity magnitude when executing characteristic stroke with pelvis actuated. (**a**) Surge vs. heave for robot and simulation. (**b**) Resultant velocity magnitude.

Similarly to the results of the characteristic stroke, when the pelvis was actuated with the characteristic stroke on both systems, the numerical model closely matched translation in surge with a larger deviation in heave. The numerical model translated 2.10 m in surge, compared to 2.09 m for the robot (~0.47% lower), while heave translation was 0.25 m for the model and negligible for the robot (Figure 11a).

The mean resultant velocity magnitude increased for every cycle for both systems. In the numerical model, mean velocity increased from 0.11 m/s to 0.27 m/s (~146%) and 0.36 m/s (~33%) (Table 2). For the robot, it rose from 0.16 m/s to 0.27 m/s (~69%) and 0.29 m/s (~7%). The robot had a ~46% higher mean in cycle 1, nearly identical mean in cycle 2, and ~24% lower mean in cycle 3.

Like the mean velocity magnitude, the maximum resultant velocity magnitude increased progressively for every cycle on both the bio-robotic system as well as the numerical model. For instance, the maximum velocity increased from 0.23 m/s to 0.35 m/s (~52%) and 0.42 m/s (~20%) for the model, and from 0.35 m/s to 0.38 m/s (~9%) for the robot (Table 2). While the robot had ~52% higher maximum velocity in cycle 1, the difference narrowed to ~8–11% in later cycles.

### 3.5. Angular Position and Angular Velocity During Characteristic Stroke with Pelvis Actuation

**Figure 12:**
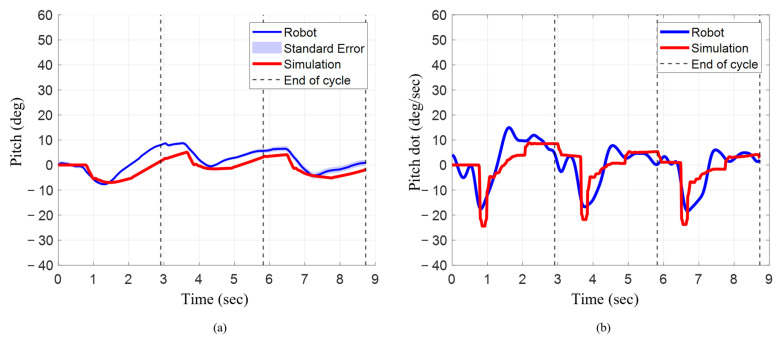
Orientation and angular rate for robot and simulation during characteristic stroke with pelvis actuated. (**a**) Pitch angle changes across three strokes. (**b**) Angular velocity across three strokes.

The final pitch angle decreased progressively after each cycle when the characteristic stroke with pelvis actuation was implemented on the bio-robotic system, but first increased and then decreased for the numerical model. In the numerical model, the final pitch angle after cycle 1 was 2° (Figure 12a). At the end of cycle 2, the final pitch angle increased by 50% to 3°. The final pitch angle decreased by about 166.70% going from cycle 2 to cycle 3 to approximately −2° (Table 3). For the robotic system, at the end of cycle 1, the final pitch angle was 8°. The final pitch angle decreased by approximately 25% and 88% in cycle 2 and cycle 3, respectively. When comparing both systems, at the end of cycles 1, 2 and 3, the numerical model was about 75%, 50%, and 300% lower, respectively.

The mean angular velocity progressively decreased for every cycle for the robotic system, but for the numerical model, it slightly increased from cycle 1 to cycle 2 and then decreased from cycle 2 to cycle 3 (Figure 12b). For the numerical model, the mean angular velocity was approximately 0.8°/s for cycle 1 and 0.9°/s for cycle 2, which was approximately 13% higher. However, going from cycle 2 to cycle 3, the mean angular velocity decreased by about 255% to −1.4°/s. For the robotic system, during cycle 1, the mean angular velocity was approximately 2.7°/s. During cycle 2, the mean angular velocity decreased by about 133% to −0.9°/s. This further decreased by about 77% to −1.6°/s. For cycle 1, the mean angular velocity of the numerical model was 66% lower. During cycle 2, the mean angular velocity for the numerical model was about 200% higher (Table 3). The mean angular velocity for the numerical model and the robotic system was much closer, with the numerical model only about 13% lower. For both the numerical model and the robotic system, the maximum angular velocity consistently decreased from cycle 1 to cycle 2 and then increased from cycle 2 to cycle 3. In the numerical model, the maximum angular velocities across each cycle were within 8% of each other. Similarly, in the robotic system, the maximum angular velocities were within 6% of each other. During cycle 1 for the numerical model, the maximum angular velocity was 24°/s. The max angular velocity decreased by 8% to 22°/s in cycle 2 and then increased back to 24°/s in cycle 3. Similarly, in the robotic system, the maximum angular velocity in cycle 1 was 18°/s. The max angular velocity decreased by 6% to 6°/s during cycle 2 and then increased back to 18°/s in cycle 3. When compared to the numerical model, the maximum angular rate for the robotic system across each cycle was about 24% lower.

### 3.6. Head and Pelvis Pitch Maneuver

**Figure 13:**
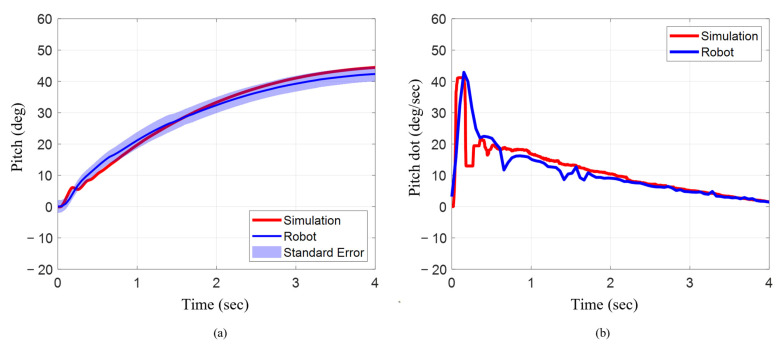
Orientation and angular rate for robot and simulation during pitch maneuver. (**a**) Pitch angle changes as head and pelvis are actuated 30° across three strokes. (**b**) Angular velocity across three strokes.

The numerical model’s prediction for the final pitch angle, as well as maximum and mean angular velocity across the four seconds during a pitch maneuver, very closely matched the bio-robotic system. The numerical model predicted a final pitch angle of 44.27° at the end of four seconds. On the other hand, the bio-robotic system produced a total pitch angle of 42.40°, which was only approximately 4% lower (Figure 13a). In addition, the maximum and mean pitch velocity across the four-second duration for the numerical model were 41.17°/s and 11.27°/s (Figure 13b). When compared to the numerical model, the maximum pitch velocity for the robot was only about 4% higher. Additionally, the mean pitch velocity for the robot was only approximately 6% lower when compared to the numerical model.

### 3.7. Head and Pelvis Yaw Maneuver

**Figure 14:**
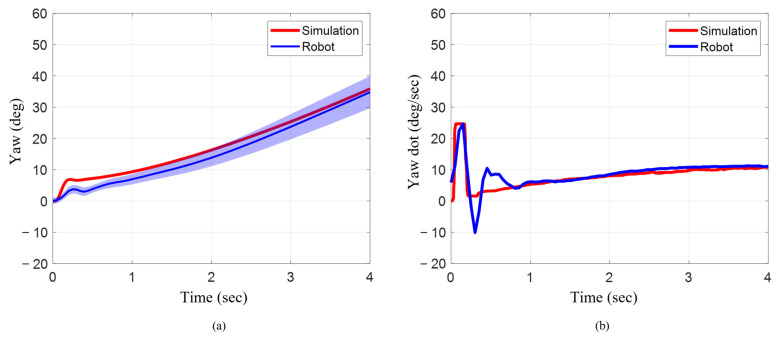
Orientation and angular rate for robot and simulation during yaw maneuver. (**a**) Pitch angle changes as head and pelvis are actuated 30° across three strokes. (**b**) Angular velocity across three strokes.

Like the pitch maneuver, when the head and pelvis were actuated for a yaw maneuver, the final yaw angle, and the maximum and mean angular velocity across the four-second duration, showed very close alignment between the numerical model and the robotic system. The final yaw angle for the numerical model at the end of four seconds was 35.84° (Figure 14a). For the robotic system, the final yaw angle was 34.80°, which was just 3% lower. The maximum and mean angular velocity across four seconds in the numerical model were 24.67°/s and 8.10°/s, respectively (Figure 14b). Similarly, the maximum angular velocity across the total duration for the robotic system was 24.63°/s, which was less than 2% lower. The mean angular velocity of the robotic system was 8.70°/s, which corresponded to an approximate increase of about 7% when compared to the numerical model. To see each of the swimming and maneuver trials executed on the bio-robotic platform and its numerical model, please refer to the following Supplementary Video S1: gavideo.

## 4. Discussion

This study successfully developed and validated a numerical model of a bio-robotic sea lion using closed-form nonlinear equations of motion. The model achieved over 99% accuracy in the prediction of surge and approximately 85% accuracy in mean angular pitch rates during swimming tests, with larger deviations observed in heave. During maneuver tests in pitch and yaw directions, where the head and pelvis were actuated simultaneously, the model closely aligned with experimental results, achieving approximately 90% accuracy in predicting rotational rates and final orientation. Crucially, the equations of motion for the model incorporated all major body segments, ensuring a comprehensive representation of the system’s dynamics. Unlike prior numerical models that are developed for uniform segmented vehicle swimming in planar motion, this numerical model captured the three-dimensional hydrodynamic forces produced by non-uniform body segments in a UUV.

Initial estimates of the hydrodynamic coefficients using traditional strip theory and assumed internal water volume resulted in limited agreement with experiments. To improve this, a genetic algorithm (GA) was employed to optimize key hydrodynamic parameters such as added mass coefficients and effective internal water volume. For example, during the execution of the characteristic stroke with pelvis actuation, the numerical model initially predicted a forward translation of 1.75 m in the X direction after three consecutive strokes. Following GA optimization, the prediction accuracy increased from approximately 84% to 99%, with the model predicting 2.1 m, which closely matched the experimental result. In the heave direction, the robot exhibited negligible vertical displacement, while the pre-GA model predicted 0.54 m of translation compared to 0.25 m after GA optimization. In terms of angular position, after three stroke cycles, the robot achieved a final orientation of 0.94°. The pre-optimization numerical model predicted 13.25°, whereas the GA-optimized model predicted −1.88°, showing a much closer agreement with the experimental result. Among all parameters considered, the internal water volume emerged as the most influential factor in improving agreement. This optimization substantially enhanced the model’s fidelity by refining the important hydrodynamic parameters to reduce the sim-to-real gap.

While the numerical model demonstrated high predictive accuracy for surge and rotational maneuvers, the primary discrepancies were observed in heave and pitch dynamics. These deviations likely resulted from differences in buoyancy distribution between the simulation and the physical robot, internal fluid movement within SEAMOUR during trials, and the influence of cable dynamics, which were not included in the simulation. In the numerical model, each body segment except the main body was assumed to be neutrally buoyant, with coincident centers of mass and buoyancy. In contrast, SEAMOUR’s head and pelvic sections, although neutrally buoyant, had non-coincident hydrostatic centers, which affected the restoring forces acting on the system. Over time, the physical robot also tended to become negatively buoyant as internal components gradually absorbed water. Furthermore, for numerical simplicity, the flippers and motors were modeled as neutrally buoyant, whereas in the physical system, they were negatively buoyant, contributing to shifts in the overall center of mass during different experimental trials. The simulation also excluded the internal movement of water, which likely varied with SEAMOUR’s speed and orientation and influenced its dynamic response. Lastly, the physical robot included a trailing antenna cable with a small surface float that was not represented in the model; however, its effect on the overall dynamics was likely marginal compared to the more significant contributions of buoyancy distribution and internal water movement.

## 5. Conclusions

In this study, a numerical model of a bio-robotic sea lion was successfully developed and validated. The model predicted the robot’s position and orientation during free-swimming and maneuver trials, demonstrating strong agreement with experimental results. The modeling framework presented in this study captured full-body kinematics of a multi-segmented system and incorporated hydrodynamic forces acting on each segment, achieving a comprehensive dynamic representation. 

The derivation of equations of motion for a multi-body UUV in closed form has potential benefits beyond the scope of this study, particularly in control theory, stability analysis, and reinforcement learning applications [21,44,45,46,47]. The closed-form equations of motion provide explicit expressions for the mass matrix, Coriolis and centripetal terms, and Generalized Force matrices that offer deep insight into the system’s inherent dynamics [22,48]. Such a formulation enables the application of advanced model-based control strategies, including feedback linearization, adaptive control, and sliding mode control [22]. Moreover, these closed-form dynamical equations facilitate the construction of Lyapunov functions, which are critical for formal stability analysis [22]. Furthermore, the model serves as an effective training environment for reinforcement learning algorithms and has previously been used to generate a novel gait for straight swimming [42].

While this current study focused on kinematic validation, future work should emphasize dynamic validation through comparison of simulated and measured actuator torques, along with the inclusion of fluid–structure interaction and internal water effects. Refining the buoyancy and mass distribution through static stability tests will further improve model fidelity. The modeling framework developed here establishes a strong foundation for extending this approach to other articulated underwater robots and for developing advanced control strategies for agile, bio-inspired underwater locomotion.

## Figures and Tables

**Table 1:** Model parameters for the numerical model.

Full model 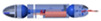	Length	L	1.12 m
	Breadth	b	0.50 m
	Width	w	0.30 m
Main body 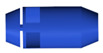	Added mass coefficients	Xudot	1.17
		Yvdot	21.26
		Zwdot	25.51
	Drag coefficients	Cdx	0.2
		Cdy	0.6
		Cdz	0.6
	Mass w/out water	mass	3.65 kg
	Inertia	(IxM, IyM, IzM)	(0.085, 0.37, 0.4)
Fore flipper 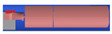	Added mass coefficients for flat plate	Ma (flipper)	0.99
	Drag coefficients for flat plate	Cd (flipper)	1.28
	Mass	mass	0.20 kg
	Inertia	(IxLF, IyLF, IzLF)	(9 × 10^−5^, 0.0013, 0.0014)
Head 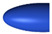	Mass w/out water	mass	2.33 kg
	Inertia	(Ixhead, Iyhead, Izhead)	(0.014, 0.045, 0.045)
Pelvis 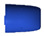	Mass w/out water	mass	1.79 kg
	Inertia	(Ixhind, Iyhind, Izhind)	(0.011, 0.039, 0.039)
Hind flipper 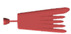	Mass	mass	0.14 kg
	Inertia	(IxLH, IyLH, IzLH)	(7 × 10^−5^, 3.94 × 10^−4^, 4.6 × 10^−4^)
Full model 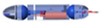	Volume of water inside SEAMOUR	volume%	40

**Table 2:** Maximum and mean resultant velocity magnitude for each cycle for robot and simulation during characteristic stroke (CS) and CS with pelvis actuation.

Velocity Magnitude	Cycle 1		Cycle 2		Cycle 3	
Stroke	Max. ||V|| (m/s)	Mean ||V|| (m/s)	Max. ||V|| (m/s)	Mean ||V|| (m/s)	Max. ||V|| (m/s)	Mean ||V|| (m/s)
CS ROBOT	0.33	0.15	0.37	0.26	0.36	0.27
CS SIM	0.21	0.09	0.32	0.24	0.39	0.32
CS with pelvis ROBOT	0.35	0.16	0.38	0.27	0.38	0.29
CS with pelvis SIM	0.22	0.11	0.35	0.27	0.42	0.36

**Table 3:** Angular position and angular velocity for each cycle for the robot and the simulation during the characteristic stroke (cs) and cs with pelvis actuation.

Orientation	Cycle 1			Cycle 2			Cycle 3		
Stroke	Final θ (deg)	Max. $\dot{\theta }$ (deg/sec)	Mean $\dot{\theta }$ (deg/sec)	Final θ (deg)	Max. $\dot{\theta }$ (deg/sec)	Mean $\dot{\theta }$ (deg/sec)	Final θ (deg)	Max. $\dot{\theta }$ (deg/sec)	Mean $\dot{\theta }$ (deg/sec)
CS ROBOT	16.89	22.62	5.81	29.43	14.85	4.31	42.16	15.16	4.82
CS SIM	12.02	10.93	4.68	30.65	8.95	6.17	35.15	4.35	1.71
CS with pelvis ROBOT	8.04	17.56	2.73	5.63	16.72	–0.86	0.94	18.43	–1.61
CS with pelvis SIM	1.58	24.39	0.81	3.15	21.77	0.87	–1.88	23.73	–1.37

## Data Availability

The data and the code used during the study are available from the corresponding author upon reasonable request.

## Supplementary Materials

The following supporting information can be downloaded at: https://www.mdpi.com/article/10.3390/biomimetics10110772/s1, Video S1: gavideo, File S1: Equations of Motion.

## Abbreviations

The following abbreviations are used in this manuscript:

UUV	Unmanned underwater vehicle
CFD	Computational fluid dynamics
DoFs	Degrees of freedom
ULL	Underwater legged locomotion
