# Supplementary material for: Development of a Numerical Model of a Bio-Inspired Sea Lion Robot
Source: Biomimetics (Basel). 2025 Nov 14;10(11):772. doi: 10.3390/biomimetics10110772 (PMC12649834; doi:10.3390/biomimetics10110772)
Supplement: Supplementary file 1 [file biomimetics-10-00772-s001.zip › biomimetics-3959602-supplementary S1.pdf]

Supplementary File (Equations of Motion)

A. Combined Equations of Motion (EoM) for multi-body system

The underlying kinematics and dynamics for the numerical model of a biologically inspired, shape-changing sea lion robot are represented:

Kinematics: $$\dot{q} = V(q)p,$$

Dynamics: $$M(q)\dot{p} + C(p,q)p + F(p,q) = Q$$

where q is a vector of all the generalized coordinates needed to represent this multi-body system. p is a vector of quasi-velocities and V(q) is the velocity transformation matrix of rank 20, which equates to 20 degrees of freedom (DoF) in this system. The generalized coordinate matrix **q** and its velocity vector **p** are shown below.

$$q = \begin{bmatrix} \text{phi} \\ \text{theta} \\ \text{psi} \\ \text{x} \\ \text{y} \\ \text{z} \\ \text{pitchLF} \\ \text{pitchRF} \\ \text{yawLF} \\ \text{yawRF} \\ \text{rollLF} \\ \text{rollRF} \\ \text{thetahead} \\ \text{psihead} \\ \text{thetahind} \\ \text{psihind} \\ \text{phiLH} \\ \text{psiLH} \\ \text{phiRH} \\ \text{psiRH} \end{bmatrix} \qquad p = \begin{bmatrix} \text{p} \\ \text{q} \\ \text{r} \\ \text{u} \\ \text{v} \\ \text{w} \\ \text{qLF} \\ \text{qRF} \\ \text{rLF} \\ \text{rRF} \\ \text{pLF} \\ \text{pRF} \\ \text{qhead} \\ \text{rhead} \\ \text{qhind} \\ \text{rhind} \\ \text{pLH} \\ \text{rLH} \\ \text{pRH} \\ \text{rRH} \end{bmatrix}$$

The mass and inertial properties for various body segments are presented in Table I of the manuscript. The mass and Coriolis matrices have a full rank of 20, which includes all degrees of freedom and body segments. Fig. A shows all variables considered to describe the geometrical features of the model. These parameters are listed in Table A1.

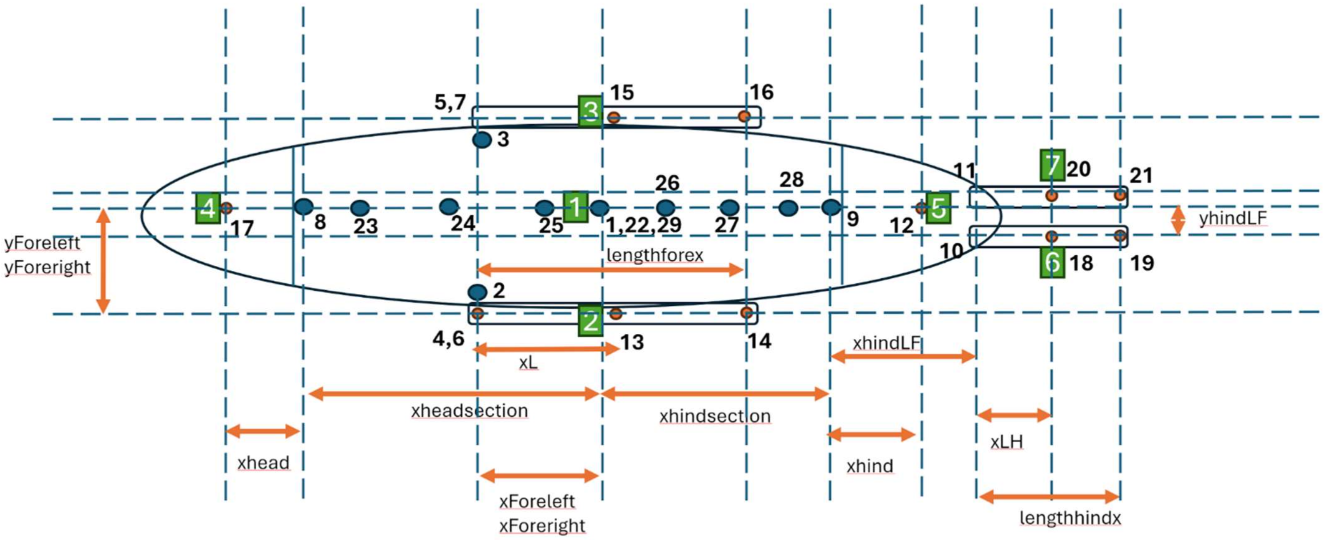

Fig. A. Schematic of the complete multi-body numerical model of SEAMOUR, illustrating all nodes and variables used to describe the system.

To represent the full body equations of motion, these matrices are decomposed into submatrices. For now, the system is in neutral configuration, where the foreflippers remain streamlined next to the main body. Additionally, to simplify the equations here, it is assumed all body segments, such as head, pelvis with hind flippers, foreflippers, and the main body, are not moving, and their respective linear and angular velocities are zero.

$$M = \begin{bmatrix} M11_{6x6} & M12_{6x14} \\ M21_{14x6} & M22_{14} \end{bmatrix}$$

$$M11_{6x6} = \begin{bmatrix} a_1 & a_2 & a_3 & 0 & a_4 & a_5 \\ a_2 & a_6 & a_7 & a_8 & 0 & a_9 \\ a_3 & a_7 & a_{10} & a_{11} & a_{12} & 0 \\ 0 & a_8 & a_{11} & a_{13} & 0 & 0 \\ a_4 & 0 & a_{12} & 0 & a_{13} & 0 \\ a_5 & a_9 & 0 & 0 & 0 & a_{13} \end{bmatrix}$$

All coefficients from the above matrix are defined below.

$$\begin{aligned} a_1 = {} & \mathrm{Ixhead} + \mathrm{Ixhind} + 2\mathrm{IxLF} + \mathrm{IxLH} + \mathrm{IxM} + 2\mathrm{Ixp} + \mathrm{IxRH} + 2\mathrm{Ixy} + \mathrm{m2yForeleft}^2 + \mathrm{m3(yFleft + yForeleft)}^2 \\ & + \mathrm{m4(yFleft + yForeleft)}^2 + \mathrm{m2yForeright}^2 + \mathrm{m3(yForeright + yFright)}^2 \\ & + \mathrm{m4(yForeright + yFright)}^2 + \mathrm{m8yhindLF}^2 + \mathrm{m9yhindRF}^2 + \mathrm{m3zFleft}^2 + \mathrm{m3zFright}^2 \\ & + \mathrm{m4(-zFleft - zleftYaw)}^2 + \mathrm{m4(-zFright - zrightYaw)}^2 \end{aligned}$$

$$
\begin{aligned}
a_2 = \frac{1}{2}\Big(&-2\mathrm{m2xForeleftyForeleft} - 2\mathrm{m3xForeleft(yFleft + yForeleft)} - \mathrm{m4xL(yFleft + yForeleft)} \\
&+ \big(-\mathrm{m4xL} + \mathrm{m4(-xForeleft - xleftYaw)}\big)\mathrm{(yFleft + yForeleft)} \\
&+ \mathrm{m4(-xForeleft - xleftYaw)(yFleft + yForeleft)}\Big) \\
&+ \frac{1}{2}\Big(-2\mathrm{m2xForerightyForeright} - 2\mathrm{m3xForeright(yForeright + yFright)} \\
&- \mathrm{m4xL(yForeright + yFright)} \\
&+ \big(-\mathrm{m4xL} + \mathrm{m4(-xForeright - xrightYaw)}\big)\mathrm{(yForeright + yFright)} \\
&+ \mathrm{m4(-xForeright - xrightYaw)(yForeright + yFright)}\Big) - \mathrm{m6xheadsectionyhead} \\
&+ \mathrm{m7xhindsectionyhind} \\
&+ \frac{1}{2}(-2\mathrm{m7xhindsectionyhind} + \mathrm{m8(-xhindLF - xhindsection)yhindLF} - \mathrm{m8xLHyhindLF} \\
&+ (\mathrm{m8(-xhindLF - xhindsection)} - \mathrm{m8xLH})\mathrm{yhindLF}) \\
&+ \frac{1}{2}(-2\mathrm{m7xhindsectionyhind} + \mathrm{m9(-xhindRF - xhindsection)yhindRF} - \mathrm{m9xRHyhindRF} \\
&+ (\mathrm{m9(-xhindRF - xhindsection)} - \mathrm{m9xRH})\mathrm{yhindRF})
\end{aligned}
$$

$$
\begin{aligned}
a_3 = &-\mathrm{m6xheadsectionzhead} - \mathrm{m7xhindsectionzhind} + \frac{1}{2}(-2\mathrm{m3xForeleftzFleft} + \mathrm{m4xL(-zFleft - zleftYaw)} \\
&+ \mathrm{m4(xForeleft + xleftYaw)(-zFleft - zleftYaw)} + (\mathrm{m4xL} + \mathrm{m4(xForeleft} \\
&+ \mathrm{xleftYaw))(-zFleft - zleftYaw))} + \frac{1}{2}(-2\mathrm{m3xForerightzFright} + \mathrm{m4xL(-zFright} \\
&- \mathrm{zrightYaw)} + \mathrm{m4(xForeright + xrightYaw)(-zFright - zrightYaw)} + (\mathrm{m4xL} \\
&+ \mathrm{m4(xForeright + xrightYaw))(-zFright - zrightYaw)))}
\end{aligned}
$$

$$
\begin{aligned}
a_4 = \mathrm{m1zg} &+ \frac{1}{2}(-2\mathrm{m1zg} - 2\mathrm{m6zhead}) - 2\mathrm{m7zhind} + \frac{1}{2}(2\mathrm{m1zg} + 2\mathrm{m7zhind}) \\
&+ \frac{1}{2}\big(-2\mathrm{m3zFleft} - 2\mathrm{m1zg} + 2\mathrm{m4(-zFleft - zleftYaw)}\big) \\
&+ \frac{1}{2}\big(-2\mathrm{m3zFright} - 2\mathrm{m1zg} + 2\mathrm{m4(-zFright - zrightYaw)}\big)
\end{aligned}
$$

$$
\begin{aligned}
a_5 = -3\mathrm{m1yg} &+ \frac{1}{2}(2\mathrm{m2yForeleft} + 2\mathrm{m3(yFleft + yForeleft)} + 2\mathrm{m4(yFleft + yForeleft)} + 2\mathrm{m1yg}) \\
&+ \frac{1}{2}(2\mathrm{m2yForeright} + 2\mathrm{m3(yForeright + yFright)} + 2\mathrm{m4(yForeright + yFright)} + 2\mathrm{m1yg}) \\
&+ \frac{1}{2}(2\mathrm{m1yg} + 2\mathrm{m6yhead}) + \frac{1}{2}(-2\mathrm{m1yg} - 2\mathrm{m7yhind}) \\
&+ \frac{1}{2}(2\mathrm{m1yg} + 2\mathrm{m7yhind} + 2\mathrm{m8yhindLF}) + \frac{1}{2}(2\mathrm{m1yg} + 2\mathrm{m7yhind} + 2\mathrm{m9yhindRF})
\end{aligned}
$$

$$
\begin{aligned}
a_6 = {} & \mathrm{Iyhead} + \mathrm{Iyhind} + 2\mathrm{IyLF} + \mathrm{IyLH} + \mathrm{IyM} + 2\mathrm{Iyp} + \mathrm{IyRH} + 2\mathrm{Iyy} + \mathrm{m2xForeleft}^2 + \mathrm{m3xForeleft}^2 \\
& + \mathrm{m2xForeright}^2 + \mathrm{m3xForeright}^2 + \mathrm{m6xheadxheadsection} \\
& - \mathrm{xheadsection}(-\mathrm{m6xhead} - \mathrm{m6xheadsection}) + \mathrm{m7xhindxhindsection} \\
& - \mathrm{xhindsection}(-\mathrm{m7xhind} - \mathrm{m7xhindsection}) - \mathrm{m4xL}(-\mathrm{xForeleft} - \mathrm{xleftYaw}) \\
& + \big(-\mathrm{m4xL} + \mathrm{m4}(-\mathrm{xForeleft} - \mathrm{xleftYaw})\big)(-\mathrm{xForeleft} - \mathrm{xleftYaw}) \\
& - \mathrm{m8}(-\mathrm{xhindLF} - \mathrm{xhindsection})\mathrm{xLH} \\
& + (-\mathrm{xhindLF} - \mathrm{xhindsection})(\mathrm{m8}(-\mathrm{xhindLF} - \mathrm{xhindsection}) - \mathrm{m8xLH}) \\
& - \mathrm{m9}(-\mathrm{xhindRF} - \mathrm{xhindsection})\mathrm{xRH} \\
& + (-\mathrm{xhindRF} - \mathrm{xhindsection})(\mathrm{m9}(-\mathrm{xhindRF} - \mathrm{xhindsection}) - \mathrm{m9xRH}) \\
& - \mathrm{m4xL}(-\mathrm{xForeright} - \mathrm{xrightYaw}) \\
& + \big(-\mathrm{m4xL} + \mathrm{m4}(-\mathrm{xForeright} - \mathrm{xrightYaw})\big)(-\mathrm{xForeright} - \mathrm{xrightYaw}) + \mathrm{m3zFleft}^2 \\
& + \mathrm{m3zFright}^2 + \mathrm{m4}(\mathrm{zFleft} + \mathrm{zleftYaw})^2 + \mathrm{m4}(\mathrm{zFright} + \mathrm{zrightYaw})^2
\end{aligned}
$$

$$
\begin{aligned}
a_7 = {} & \frac{1}{2}\big(2\mathrm{m3}(-\mathrm{yFleft} - \mathrm{yForeleft})\mathrm{zFleft} + 2\mathrm{m4}(-\mathrm{yFleft} - \mathrm{yForeleft})(\mathrm{zFleft} + \mathrm{zleftYaw})\big) \\
& + \frac{1}{2}\big(2\mathrm{m3}(-\mathrm{yForeright} - \mathrm{yFright})\mathrm{zFright} \\
& + 2\mathrm{m4}(-\mathrm{yForeright} - \mathrm{yFright})(\mathrm{zFright} + \mathrm{zrightYaw})\big)
\end{aligned}
$$

$$
\begin{aligned}
a_8 = {} & -\mathrm{m1zg} + \frac{1}{2}(2\mathrm{m1zg} + 2\mathrm{m6zhead}) + 2\mathrm{m7zhind} + \frac{1}{2}(-2\mathrm{m1zg} - 2\mathrm{m7zhind}) \\
& + \frac{1}{2}\big(2\mathrm{m3zFleft} + 2\mathrm{m1zg} + 2\mathrm{m4}(\mathrm{zFleft} + \mathrm{zleftYaw})\big) \\
& + \frac{1}{2}\big(2\mathrm{m3zFright} + 2\mathrm{m1zg} + 2\mathrm{m4}(\mathrm{zFright} + \mathrm{zrightYaw})\big)
\end{aligned}
$$

$$
\begin{aligned}
a_9 = {} & 3\mathrm{m1xg} + \frac{1}{2}(-2\mathrm{m1xg} - 2\mathrm{m6xhead} - 2\mathrm{m6xheadsection}) + \frac{1}{2}(2\mathrm{m1xg} + 2\mathrm{m7xhind} + 2\mathrm{m7xhindsection}) \\
& + \frac{1}{2}\big(-2\mathrm{m2xForeleft} - 2\mathrm{m3xForeleft} - 2\mathrm{m1xg} - 2\mathrm{m4xL} + 2\mathrm{m4}(-\mathrm{xForeleft} - \mathrm{xleftYaw})\big) \\
& + \frac{1}{2}(-2\mathrm{m1xg} - 2\mathrm{m7xhind} + 2\mathrm{m8}(-\mathrm{xhindLF} - \mathrm{xhindsection}) - 2\mathrm{m7xhindsection} \\
& - 2\mathrm{m8xLH}) \\
& + \frac{1}{2}(-2\mathrm{m1xg} - 2\mathrm{m7xhind} + 2\mathrm{m9}(-\mathrm{xhindRF} - \mathrm{xhindsection}) - 2\mathrm{m7xhindsection} \\
& - 2\mathrm{m9xRH}) \\
& + \frac{1}{2}\big(-2\mathrm{m2xForeright} - 2\mathrm{m3xForeright} - 2\mathrm{m1xg} - 2\mathrm{m4xL} \\
& + 2\mathrm{m4}(-\mathrm{xForeright} - \mathrm{xrightYaw})\big)
\end{aligned}
$$

$$\begin{aligned}
\mathrm{a}_{10} = {} & \mathrm{Izhead + Izhind + 2IzLF + IzLH + IzM + 2Izp + IzRH + 2Izy + m2xForeleft^2 + m3xForeleft^2} \\
& \mathrm{+ m2xForeright^2 + m3xForeright^2 + m6xheadxheadsection} \\
& \mathrm{+ xheadsection(m6xhead + m6xheadsection) + m7xhindxhindsection} \\
& \mathrm{+ xhindsection(m7xhind + m7xhindsection) + m4xL(xForeleft + xleftYaw)} \\
& \mathrm{+ (xForeleft + xleftYaw)\big(m4xL + m4(xForeleft + xleftYaw)\big)} \\
& \mathrm{+ m8(xhindLF + xhindsection)xLH} \\
& \mathrm{+ (xhindLF + xhindsection)(m8(xhindLF + xhindsection) + m8xLH)} \\
& \mathrm{+ m9(xhindRF + xhindsection)xRH} \\
& \mathrm{+ (xhindRF + xhindsection)(m9(xhindRF + xhindsection) + m9xRH)} \\
& \mathrm{+ m4xL(xForeright + xrightYaw)} \\
& \mathrm{+ (xForeright + xrightYaw)\big(m4xL + m4(xForeright + xrightYaw)\big)} \\
& \mathrm{+ m3(-yFleft - yForeleft)^2 + m4(-yFleft - yForeleft)^2 + m2yForeleft^2 + m2yForeright^2} \\
& \mathrm{+ m3(-yForeright - yFright)^2 + m4(-yForeright - yFright)^2 + m8yhindLF^2} \\
& \mathrm{+ m9yhindRF^2}
\end{aligned}$$

$$\begin{aligned}
\mathrm{a}_{11} = {} & \mathrm{3m1yg} + \frac{1}{2}\mathrm{(2m3(-yFleft - yForeleft) + 2m4(-yFleft - yForeleft) - 2m2yForeleft - 2m1yg)} \\
& + \frac{1}{2}\mathrm{(-2m2yForeright + 2m3(-yForeright - yFright) + 2m4(-yForeright - yFright)} \\
& \mathrm{- 2m1yg)} + \frac{1}{2}\mathrm{(-2m1yg - 2m6yhead)} + \frac{1}{2}\mathrm{(2m1yg + 2m7yhind)} \\
& + \frac{1}{2}\mathrm{(-2m1yg - 2m7yhind - 2m8yhindLF)} + \frac{1}{2}\mathrm{(-2m1yg - 2m7yhind - 2m9yhindRF)}
\end{aligned}$$

$$\begin{aligned}
\mathrm{a}_{12} = {} & \mathrm{-3m1xg} + \frac{1}{2}\mathrm{(2m1xg + 2m6xhead + 2m6xheadsection)} \\
& + \frac{1}{2}\mathrm{(-2m1xg - 2m7xhind - 2m7xhindsection)} \\
& + \frac{1}{2}\mathrm{\big(2m2xForeleft + 2m3xForeleft + 2m1xg + 2m4xL + 2m4(xForeleft + xleftYaw)\big)} \\
& + \frac{1}{2}\mathrm{(2m1xg + 2m7xhind + 2m7xhindsection + 2m8(xhindLF + xhindsection) + 2m8xLH)} \\
& + \frac{1}{2}\mathrm{(2m1xg + 2m7xhind + 2m7xhindsection + 2m9(xhindRF + xhindsection) + 2m9xRH)} \\
& + \frac{1}{2}\mathrm{\big(2m2xForeright + 2m3xForeright + 2m1xg + 2m4xL + 2m4(xForeright + xrightYaw)\big)}
\end{aligned}$$

$$\mathrm{a}_{13} = \mathrm{m1 + 2m2 + 2m3 + 2m4 + m6 + m7 + m8 + m9}$$

$$\mathrm{M12}_{6x14} = \begin{bmatrix}
b_1 & b_6 & b_{11} & b_{14} & \mathrm{IxLF} & \mathrm{IxLF} & 0 & 0 & b_{21} & 0 & \mathrm{IxLH} & 0 & \mathrm{IxRH} & 0 \\
b_2 & b_7 & 0 & 0 & 0 & 0 & b_{17} & 0 & b_{22} & 0 & 0 & 0 & 0 & 0 \\
b_3 & b_8 & b_{12} & b_{15} & 0 & 0 & 0 & b_{18} & 0 & b_{25} & 0 & b_{28} & 0 & b_{29} \\
0 & 0 & 0 & 0 & 0 & 0 & b_{19} & -\mathrm{m6.yhead} & b_{23} & b_{26} & 0 & 0 & 0 & 0 \\
b_4 & b_9 & b_{13} & b_{16} & 0 & 0 & 0 & \mathrm{m6.xhead} & 0 & b_{27} & 0 & \mathrm{m8.xLH} & 0 & \mathrm{m9.xRH} \\
b_5 & b_{10} & 0 & 0 & 0 & 0 & b_{20} & 0 & b_{24} & 0 & 0 & 0 & 0 & 0
\end{bmatrix}$$

All coefficients from the above matrix are defined below.

$$\begin{aligned}
\mathrm{b}_1 = {} & \frac{1}{2}\mathrm{(2IxLF + 2Ixp + 2Ixy + 2m3yFleft(yFleft + yForeleft) + 2m4yFleft(yFleft + yForeleft) + 2m3zFleft^2} \\
& \mathrm{+ 2m4(-zFleft - zleftYaw)^2)}
\end{aligned}$$

$$b_2 = \frac{1}{2}(-2m3xForeleftyFleft - m4xLyFleft + (-m4xL + m4(-xForeleft - xleftYaw))yFleft + m4(-xForeleft - xleftYaw)yFleft)$$

$$b_3 = \frac{1}{2}(-2m3xForeleftzFleft + m4xL(-zFleft - zleftYaw) + m4(xForeleft + xleftYaw)(-zFleft - zleftYaw) + (m4xL + m4(xForeleft + xleftYaw))(-zFleft - zleftYaw))$$

$$b_4 = \frac{1}{2}(-2m3zFleft + 2m4(-zFleft - zleftYaw))$$

$$b_5 = \frac{1}{2}(2m3yFleft + 2m4yFleft)$$

$$b_6 = \frac{1}{2}(2IxLF + 2Ixp + 2Ixy + 2m3yFright(yForeright + yFright) + 2m4yFright(yForeright + yFright) + 2m3zFright^2 + 2m4(-zFright - zrightYaw)^2)$$

$$b_7 = \frac{1}{2}(-2m3xForerightyFright - m4xLyFright + (-m4xL + m4(-xForeright - xrightYaw))yFright + m4(-xForeright - xrightYaw)yFright)$$

$$b_8 = \frac{1}{2}(-2m3xForerightzFright + m4xL(-zFright - zrightYaw) + m4(xForeright + xrightYaw)(-zFright - zrightYaw) + (m4xL + m4(xForeright + xrightYaw))(-zFright - zrightYaw))$$

$$b_9 = \frac{1}{2}(-2m3zFright + 2m4(-zFright - zrightYaw))$$

$$b_{10} = \frac{1}{2}(2m3yFright + 2m4yFright)$$

$$b_{11} = \frac{1}{2}(m4xL(-zFright - zrightYaw) + m4xrightYaw(-zFright - zrightYaw) + (m4xL + m4xrightYaw)(-zFright - zrightYaw))$$

$$b_{12} = \frac{1}{2}(2IzLF + 2Izy + m4xLxrightYaw + m4xL(xForeright + xrightYaw) + (xForeright + xrightYaw)(m4xL + m4xrightYaw) + xrightYaw(m4xL + m4(xForeright + xrightYaw)))$$

$$b_{13} = \frac{1}{2}(2m4xL + 2m4xrightYaw)$$

$$b_{14} = \frac{1}{2}(m4xL(-zFright - zrightYaw) + m4xrightYaw(-zFright - zrightYaw) + (m4xL + m4xrightYaw)(-zFright - zrightYaw))$$

$$b_{15} = \frac{1}{2}(2IzLF + 2Izy + m4xLxrightYaw + m4xL(xForeright + xrightYaw) + (xForeright + xrightYaw)(m4xL + m4xrightYaw) + xrightYaw(m4xL + m4(xForeright + xrightYaw)))$$

$$b_{16} = \frac{1}{2}(2m4xL + 2m4xrightYaw)$$

$$b_{17} = Iyhead + m6xheadxheadsection$$

$$b_{18} = Izhead + m6xheadxheadsection$$

$$b_{19} = m6.\,zhead$$

$$b_{20} = -m6.\,xhead$$

$$b_{21} = \frac{1}{2}(-\text{m8xhindLFyhindLF} - \text{m8xLHyhindLF} + (-\text{m8xhindLF} - \text{m8xLH})\text{yhindLF}) + \frac{1}{2}(-\text{m9xhindRFyhindRF} - \text{m9xRHyhindRF} + (-\text{m9xhindRF} - \text{m9xRH})\text{yhindRF})$$

$$b_{22} = -\text{Iyhind} - \text{m7xhindxhindsection} + \frac{1}{2}(2\text{Iyhind} + 2\text{IyLH} + 2\text{m7xhindxhindsection} + \text{m8xhindLFxLH} - \text{m8}(-\text{xhindLF} - \text{xhindsection})\text{xLH} + (-\text{xhindLF} - \text{xhindsection})(-\text{m8xhindLF} - \text{m8xLH}) - \text{xhindLF}(\text{m8}(-\text{xhindLF} - \text{xhindsection}) - \text{m8xLH})) + \frac{1}{2}(2\text{Iyhind} + 2\text{IyRH} + 2\text{m7xhindxhindsection} + \text{m9xhindRFxRH} - \text{m9}(-\text{xhindRF} - \text{xhindsection})\text{xRH} + (-\text{xhindRF} - \text{xhindsection})(-\text{m9xhindRF} - \text{m9xRH}) - \text{xhindRF}(\text{m9}(-\text{xhindRF} - \text{xhindsection}) - \text{m9xRH}))$$

$$b_{23} = \text{m7zhind}$$

$$b_{24} = \text{m7xhind} + \frac{1}{2}(-2\text{m7xhind} - 2\text{m8xhindLF} - 2\text{m8xLH}) + \frac{1}{2}(-2\text{m7xhind} - 2\text{m9xhindRF} - 2\text{m9xRH})$$

$$b_{25} = -\text{Izhind} - \text{m7xhindxhindsection} + \frac{1}{2}(2\text{Izhind} + 2\text{IzLH} + 2\text{m7xhindxhindsection} + \text{m8xhindLFxLH} + \text{m8}(\text{xhindLF} + \text{xhindsection})\text{xLH} + (\text{xhindLF} + \text{xhindsection})(\text{m8xhindLF} + \text{m8xLH}) + \text{xhindLF}(\text{m8}(\text{xhindLF} + \text{xhindsection}) + \text{m8xLH}) + 2\text{m8yhindLF}^2) + \frac{1}{2}(2\text{Izhind} + 2\text{IzRH} + 2\text{m7xhindxhindsection} + \text{m9xhindRFxRH} + \text{m9}(\text{xhindRF} + \text{xhindsection})\text{xRH} + (\text{xhindRF} + \text{xhindsection})(\text{m9xhindRF} + \text{m9xRH}) + \text{xhindRF}(\text{m9}(\text{xhindRF} + \text{xhindsection}) + \text{m9xRH}) + 2\text{m9yhindRF}^2)$$

$$b_{26} = \text{m7yhind} + \frac{1}{2}(-2\text{m7yhind} - 2\text{m8yhindLF}) + \frac{1}{2}(-2\text{m7yhind} - 2\text{m9yhindRF})$$

$$b_{27} = -\text{m7xhind} + \frac{1}{2}(2\text{m7xhind} + 2\text{m8xhindLF} + 2\text{m8xLH}) + \frac{1}{2}(2\text{m7xhind} + 2\text{m9xhindRF} + 2\text{m9xRH})$$

$$b_{28} = \text{IzLH} + \text{m8}(\text{xhindLF} + \text{xhindsection})\text{xLH}$$

$$b_{29} = \text{IzRH} + \text{m9}(\text{xhindRF} + \text{xhindsection})\text{xRH}$$

$$\text{M21}_{14\text{x}6} = \begin{bmatrix} c_1 & c_6 & c_{10} & 0 & c_{19} & c_{24} \\ c_2 & c_7 & c_{11} & 0 & c_{20} & c_{25} \\ c_3 & 0 & c_{12} & 0 & c_{21} & 0 \\ c_4 & 0 & c_{13} & 0 & c_{22} & 0 \\ \text{IxLF} & 0 & 0 & 0 & 0 & 0 \\ \text{IxLF} & 0 & 0 & 0 & 0 & 0 \\ 0 & c_8 & 0 & \text{m6.zhead} & 0 & -\text{m6.xhead} \\ 0 & 0 & c_{14} & -\text{m6.yhead} & \text{m6.xhead} & 0 \\ c_5 & c_9 & 0 & \text{m7.zhind} & 0 & c_{26} \\ 0 & 0 & c_{15} & c_{18} & c_{23} & 0 \\ \text{IxLH} & 0 & 0 & 0 & 0 & 0 \\ 0 & 0 & c_{16} & 0 & \text{m8.xLH} & 0 \\ \text{IxRH} & 0 & 0 & 0 & 0 & 0 \\ 0 & 0 & c_{17} & 0 & \text{m9.xRH} & 0 \end{bmatrix}$$

All coefficients from the above matrix are defined below.

$$c_1 = \frac{1}{2}(2IxLF + 2Ixp + 2Ixy + 2m3yFleft(yFleft + yForeleft) + 2m4yFleft(yFleft + yForeleft) + 2m3zFleft^2 + 2m4(-zFleft - zleftYaw)^2)$$

$$c_2 = \frac{1}{2}(2IxLF + 2Ixp + 2Ixy + 2m3yFright(yForeright + yFright) + 2m4yFright(yForeright + yFright) + 2m3zFright^2 + 2m4(-zFright - zrightYaw)^2)$$

$$c_3 = \frac{1}{2}(m4xL(-zFleft - zleftYaw) + m4xleftYaw(-zFleft - zleftYaw) + (m4xL + m4xleftYaw)(-zFleft - zleftYaw))$$

$$c_4 = \frac{1}{2}(m4xL(-zFright - zrightYaw) + m4xrightYaw(-zFright - zrightYaw) + (m4xL + m4xrightYaw)(-zFright - zrightYaw))$$

$$c_5 = \frac{1}{2}(-m8xhindLFyhindLF - m8xLHyhindLF + (-m8xhindLF - m8xLH)yhindLF) + \frac{1}{2}(-m9xhindRFyhindRF - m9xRHyhindRF + (-m9xhindRF - m9xRH)yhindRF)$$

$$c_6 = \frac{1}{2}(-2m3xForeleftyFleft - m4xLyFleft + (-m4xL + m4(-xForeleft - xleftYaw))yFleft + m4(-xForeleft - xleftYaw)yFleft)$$

$$c_7 = \frac{1}{2}(-2m3xForerightyFright - m4xLyFright + (-m4xL + m4(-xForeright - xrightYaw))yFright + m4(-xForeright - xrightYaw)yFright)$$

$$c_8 = Iyhead + m6xheadxheadsection$$

$$c_9 = -Iyhind - m7xhindxhindsection + \frac{1}{2}(2Iyhind + 2IyLH + 2m7xhindxhindsection + m8xhindLFxLH - m8(-xhindLF - xhindsection)xLH + (-xhindLF - xhindsection)(-m8xhindLF - m8xLH) - xhindLF(m8(-xhindLF - xhindsection) - m8xLH)) + \frac{1}{2}(2Iyhind + 2IyRH + 2m7xhindxhindsection + m9xhindRFxRH - m9(-xhindRF - xhindsection)xRH + (-xhindRF - xhindsection)(-m9xhindRF - m9xRH) - xhindRF(m9(-xhindRF - xhindsection) - m9xRH))$$

$$c_{10} = \frac{1}{2}(-2m3xForeleftzFleft + m4xL(-zFleft - zleftYaw) + m4(xForeleft + xleftYaw)(-zFleft - zleftYaw) + (m4xL + m4(xForeleft + xleftYaw))(-zFleft - zleftYaw))$$

$$c_{11} = \frac{1}{2}(-2m3xForerightzFright + m4xL(-zFright - zrightYaw) + m4(xForeright + xrightYaw)(-zFright - zrightYaw) + (m4xL + m4(xForeright + xrightYaw))(-zFright - zrightYaw))$$

$$c_{12} = \frac{1}{2}(2IzLF + 2Izy + m4xLxleftYaw + m4xL(xForeleft + xleftYaw) + (xForeleft + xleftYaw)(m4xL + m4xleftYaw) + xleftYaw(m4xL + m4(xForeleft + xleftYaw)))$$

$$c_{13} = \frac{1}{2}(2IzLF + 2Izy + m4xLxrightYaw + m4xL(xForeright + xrightYaw) + (xForeright + xrightYaw)(m4xL + m4xrightYaw) + xrightYaw(m4xL + m4(xForeright + xrightYaw)))$$

$$c_{14} = Izhead + m6xheadxheadsection$$

$$c_{15} = -\mathrm{Izhind} - \mathrm{m7xhindxhindsection} + \frac{1}{2}(2\mathrm{Izhind} + 2\mathrm{IzLH} + 2\mathrm{m7xhindxhindsection} + \mathrm{m8xhindLFxLH} + \mathrm{m8}(\mathrm{xhindLF} + \mathrm{xhindsection})\mathrm{xLH} + (\mathrm{xhindLF} + \mathrm{xhindsection})(\mathrm{m8xhindLF} + \mathrm{m8xLH}) + \mathrm{xhindLF}(\mathrm{m8}(\mathrm{xhindLF} + \mathrm{xhindsection}) + \mathrm{m8xLH}) + 2\mathrm{m8yhindLF}^2) + \frac{1}{2}(2\mathrm{Izhind} + 2\mathrm{IzRH} + 2\mathrm{m7xhindxhindsection} + \mathrm{m9xhindRFxRH} + \mathrm{m9}(\mathrm{xhindRF} + \mathrm{xhindsection})\mathrm{xRH} + (\mathrm{xhindRF} + \mathrm{xhindsection})(\mathrm{m9xhindRF} + \mathrm{m9xRH}) + \mathrm{xhindRF}(\mathrm{m9}(\mathrm{xhindRF} + \mathrm{xhindsection}) + \mathrm{m9xRH}) + 2\mathrm{m9yhindRF}^2)$$

$$c_{16} = \mathrm{IzLH} + \mathrm{m8}(\mathrm{xhindLF} + \mathrm{xhindsection})\mathrm{xLH}$$

$$c_{17} = \mathrm{IzRH} + \mathrm{m9}(\mathrm{xhindRF} + \mathrm{xhindsection})\mathrm{xRH}$$

$$c_{18} = \mathrm{m7yhind} + \frac{1}{2}(-2\mathrm{m7yhind} - 2\mathrm{m8yhindLF}) + \frac{1}{2}(-2\mathrm{m7yhind} - 2\mathrm{m9yhindRF})$$

$$c_{19} = \frac{1}{2}(-2\mathrm{m3zFleft} + 2\mathrm{m4}(-\mathrm{zFleft} - \mathrm{zleftYaw}))$$

$$c_{20} = \frac{1}{2}(-2\mathrm{m3zFright} + 2\mathrm{m4}(-\mathrm{zFright} - \mathrm{zrightYaw}))$$

$$c_{21} = \frac{1}{2}(2\mathrm{m4xL} + 2\mathrm{m4xleftYaw})$$

$$c_{22} = \frac{1}{2}(2\mathrm{m4xL} + 2\mathrm{m4xrightYaw})$$

$$c_{23} = -\mathrm{m7xhind} + \frac{1}{2}(2\mathrm{m7xhind} + 2\mathrm{m8xhindLF} + 2\mathrm{m8xLH}) + \frac{1}{2}(2\mathrm{m7xhind} + 2\mathrm{m9xhindRF} + 2\mathrm{m9xRH})$$

$$c_{24} = \frac{1}{2}(2\mathrm{m3yFleft} + 2\mathrm{m4yFleft})$$

$$c_{25} = \frac{1}{2}(2\mathrm{m3yFright} + 2\mathrm{m4yFright})$$

$$c_{26} = \mathrm{m7xhind} + \frac{1}{2}(-2\mathrm{m7xhind} - 2\mathrm{m8xhindLF} - 2\mathrm{m8xLH}) + \frac{1}{2}(-2\mathrm{m7xhind} - 2\mathrm{m9xhindRF} - 2\mathrm{m9xRH})$$

$$\mathrm{M22}_{14x14} = \begin{bmatrix} d_1 & 0 & d_2 & 0 & \mathrm{IxLF} & 0 & 0 & 0 & 0 & 0 & 0 & 0 & 0 & 0 \\ 0 & d_3 & 0 & d_4 & 0 & \mathrm{IxLF} & 0 & 0 & 0 & 0 & 0 & 0 & 0 & 0 \\ d_2 & 0 & d_5 & 0 & 0 & 0 & 0 & 0 & 0 & 0 & 0 & 0 & 0 & 0 \\ 0 & d_4 & 0 & d_6 & 0 & 0 & 0 & 0 & 0 & 0 & 0 & 0 & 0 & 0 \\ \mathrm{IxLF} & 0 & 0 & 0 & \mathrm{IxLF} & 0 & 0 & 0 & 0 & 0 & 0 & 0 & 0 & 0 \\ 0 & \mathrm{IxLF} & 0 & 0 & 0 & \mathrm{IxLF} & 0 & 0 & 0 & 0 & 0 & 0 & 0 & 0 \\ 0 & 0 & 0 & 0 & 0 & 0 & \mathrm{Iyhead} & 0 & 0 & 0 & 0 & 0 & 0 & 0 \\ 0 & 0 & 0 & 0 & 0 & 0 & 0 & \mathrm{Izhead} & 0 & 0 & 0 & 0 & 0 & 0 \\ 0 & 0 & 0 & 0 & 0 & 0 & 0 & 0 & d_7 & 0 & 0 & 0 & 0 & 0 \\ 0 & 0 & 0 & 0 & 0 & 0 & 0 & 0 & 0 & d_8 & 0 & d_9 & 0 & d_{10} \\ 0 & 0 & 0 & 0 & 0 & 0 & 0 & 0 & 0 & 0 & \mathrm{IxLH} & 0 & 0 & 0 \\ 0 & 0 & 0 & 0 & 0 & 0 & 0 & 0 & 0 & d_9 & 0 & \mathrm{IzLH} & 0 & 0 \\ 0 & 0 & 0 & 0 & 0 & 0 & 0 & 0 & 0 & 0 & 0 & 0 & \mathrm{IxRH} & 0 \\ 0 & 0 & 0 & 0 & 0 & 0 & 0 & 0 & 0 & d_{10} & 0 & 0 & 0 & \mathrm{IzRH} \end{bmatrix}$$

All coefficients from the above matrix are defined below.

$$d_1 = \mathrm{IxLF} + \mathrm{Ixp} + \mathrm{Ixy} + \mathrm{m3yFleft}^2 + \mathrm{m4yFleft}^2 + \mathrm{m3zFleft}^2 + \mathrm{m4}(-\mathrm{zFleft} - \mathrm{zleftYaw})^2$$

$$d_2 = \frac{1}{2}\big(\text{m4xL}(-\text{zFleft} - \text{zleftYaw}) + \text{m4xleftYaw}(-\text{zFleft} - \text{zleftYaw}) + (\text{m4xL} + \text{m4xleftYaw})(-\text{zFleft} - \text{zleftYaw})\big)$$

$$d_3 = \text{IxLF} + \text{Ixp} + \text{Ixy} + \text{m3yFright}^2 + \text{m4yFright}^2 + \text{m3zFright}^2 + \text{m4}(-\text{zFright} - \text{zrightYaw})^2$$

$$d_4 = \frac{1}{2}\big(\text{m4xL}(-\text{zFright} - \text{zrightYaw}) + \text{m4xrightYaw}(-\text{zFright} - \text{zrightYaw}) + (\text{m4xL} + \text{m4xrightYaw})(-\text{zFright} - \text{zrightYaw})\big)$$

$$d_5 = \text{IzLF} + \text{Izy} + \text{m4xLxleftYaw} + \text{xleftYaw}(\text{m4xL} + \text{m4xleftYaw})$$

$$d_6 = \text{IzLF} + \text{Izy} + \text{m4xLxrightYaw} + \text{xrightYaw}(\text{m4xL} + \text{m4xrightYaw})$$

$$d_7 = \text{Iyhind} + \text{IyLH} + \text{IyRH} + \text{m8xhindLFxLH} - \text{xhindLF}(-\text{m8xhindLF} - \text{m8xLH}) + \text{m9xhindRFxRH} - \text{xhindRF}(-\text{m9xhindRF} - \text{m9xRH})$$

$$d_8 = \text{Izhind} + \text{IzLH} + \text{IzRH} + \text{m8xhindLFxLH} + \text{xhindLF}(\text{m8xhindLF} + \text{m8xLH}) + \text{m9xhindRFxRH} + \text{xhindRF}(\text{m9xhindRF} + \text{m9xRH}) + \text{m8yhindLF}^2 + \text{m9yhindRF}^2$$

$$d_9 = \text{IzLH} + \text{m8xhindLFxLH}$$

It is important to note that the above full mass matrix of rank 20 is symmetric. Additionally, the Coriolis and centripetal matrix typically depend on the linear and angular velocities of the generalized coordinates. Since we assumed these state variables are zero in the above displayed equations of motion, the centripetal matrix is zero. Full-ranked mass and Coriolis matrices can be seen in the full Mathematica file upon reasonable request to the authors. To better understand how this modeling approach was used to generate this comprehensive representation of multi-body equations of motion, please refer to [22].

TABLE AI

GEOMETRICAL PROPERTIES FOR VARIOUS BODY SEGMENTS

No.	Parameters	Value (m)
1	xhead	0.10
2	yhead	0.00
3	zhead	0.00
4	xg	0.00
5	yg	0.00
6	zg	0.00
7	lengthforex	0.27
8	yFleft	0.02
9	zFleft	0.01
10	xleftYaw	0.02
11	zleftYaw	0.02
12	xhind	-0.09
13	yhind	0.00
14	zhind	0.00
15	lengthhindx	0.19
16	xrightYaw	0.02
17	zrightYaw	0.02
18	yForeleft	-0.13
19	yForeright	0.13
20	yFright	0.02
21	yhindLF	0.05
22	yhindRF	-0.05
23	zFright	0.01
24	xForeleft	0.10
25	xForeright	0.10
26	xhindsection	-0.40
27	xLH	-0.09
28	xRH	-0.09
29	xL	-0.21
30	xhindLF	-0.20
31	xhindRF	-0.20
32	xheadsection	0.23
